# The Incidence of Sports-Related Concussion in Children and Adolescents: A Systematic Review and Meta-Analysis

**DOI:** 10.1186/s40798-025-00834-9

**Published:** 2025-04-11

**Authors:** Veronica Ingram, Megan Fielding, Laura A. M. Dunne, Stefan Piantella, Jonathon Weakley, Rich D. Johnston, Thomas B. McGuckian

**Affiliations:** 1 School of Behavioural and Health Sciences, Australian Catholic University, 115 Victoria Parade, Fitzroy, Melbourne, VIC 3065 Australia; 2 Healthy Brain and Mind Research Centre (HBMRC), Australian Catholic University, Melbourne, Australia; 3 Sports Performance, Recovery, Injury and New Technologies (SPRINT) Research Centre, Australian Catholic University, Melbourne, Australia; 4 Carnegie Applied Rugby Research (CARR) Centre, Carnegie School of Sport, Leeds Beckett University, Leeds, UK

**Keywords:** Youth, Athlete, SRC, Prevalence, Injury

## Abstract

**Background:**

Sport-related concussions (SRC) are a concern for young athletes due to the potential for long-term health problems. This systematic review and meta-analysis aimed to provide a comprehensive overview of the literature exploring SRC incidence in youth sports to understand the associated risks.

**Methods:**

Medline, Embase, SPORTDiscus, PsycINFO, and Web of Science databases were searched without language restrictions up to September 2024. Studies were included if they (i) reported data for calculation of SRC incidence, (ii) were a prospective cohort study, and (iii) included a sample aged ≤ 18 years. Studies that reported Athlete Exposure (AE) or Player Hours (PH) as SRC incidence data measures were included in a multi-level random-effects meta-analysis. Additional analysis explored SRC incidence based on age, sex, country, year of data collection, setting, and level of contact.

**Results:**

Of the 6474 studies reviewed for eligibility, 116 studies were accepted for a systematic review and 99 in the meta-analysis. A total of 3,025,911 participants were included in the review (59% male, 41% female); however, 41% of studies did not report sample size. The pooled incidence rate of SRC per 1000 AE was found to be 1.41 across 21 sports, and 4.36 per 1000 PH across 7 sports. The highest incidence per 1000 AE were in taekwondo, rugby union, and ice hockey, and the highest incidence per 1000 PH were in rugby 7s, rugby league, and rugby union.

**Conclusions:**

This systematic review and meta-analysis can serve as an updated baseline for risk of concussion among youth athletes across various sports. *Trial registration*: This systematic review was registered on OSF Registries (https://osf.io/v298s).

**Supplementary Information:**

The online version contains supplementary material available at 10.1186/s40798-025-00834-9.

## Key points

- Collision sports such as rugby codes, American Football and ice hockey continue to put youth athletes at a much higher risk of concussion than sports with lower levels of contact.
- Sport organisations and governing bodies are encouraged to use estimated exposure measurement as a cost effective and time efficient method of understanding concussion incidence in their specific setting.

**Supplementary Information:**

The online version contains supplementary material available at 10.1186/s40798-025-00834-9.

## Background

Sport related concussions (SRC) and repeated head impacts have become a significant concern and garnered increased attention due to the risk of long-term problems in athletes [1–3]. SRC is defined as a traumatic brain injury that is caused by a direct blow to the head, neck, or body (e.g., whiplash mechanism) resulting in an impulsive force being transmitted to the brain during sport and exercise activities [4]. Initial symptoms of concussion may include headaches, dizziness, vomiting, and nausea. Due to the potential negative outcomes of concussion, there has been an increase in the awareness [5], identification [6, 7], and diagnosis [8] of SRC. Consequently, there has been an increase in the literature regarding the prevalence and incidence of SRC.

Compared with adults, children and adolescents are more susceptible to concussions because of a higher vulnerability of the developing brain, weaker neck muscles, and larger head to body ratio [9, 10]. The prefrontal cortex, which is responsible for executive functions, does not fully develop until early adulthood and is particularly vulnerable to injury during adolescence [11]. As a result, paediatric populations can experience a range of serious short- and long-term symptoms of concussions that have the potential to impact development [12]. Common symptoms include fatigue, headaches, and loss of concentration [13, 14]. In addition, concussive injuries in children may also lead to sleep disturbances, future limitations in physical activity, and vision and hearing challenges [15]. These symptoms can consequently have a negative effect on a young person’s academic performance and learning [16]. Concussions may also substantially impact young people’s emotional development, whereby individuals may be at increased risk of developing mental health problems such as heightened attentional problems, anxiety, and depression [17]. These changes can have a considerable effect on relationships youth share with their peers and family [18]. Finally, repetitive trauma through concussive and non-concussive head impacts may be a likely link in the development of neurodegenerative conditions (e.g., chronic traumatic encephalopathy (CTE) [3]. Therefore, given the potential impact of youth concussions, it is important to understand the incidence of SRC within child and adolescent populations.

Children and adolescents participate in sports more than adults [19], but there is concern regarding the underreporting of concussions in youth sports [20, 21]. Children who have had a previous history of SRC are at an increased risk of future concussion [22], experience negative symptoms for a prolonged period [23], and often take longer to recover compared to adults [8]. Youth sports have increasingly become more physically intense as competition heightens [24, 25]. However, there has been limited evaluation of SRC injury incidence in youth sports in comparison to collegiate and professional adult athletes [26–29].

The most recent systematic review investigating SRC incidence in youth populations found the sports with the highest SRC incidence rates were rugby codes, ice hockey, and American Football [30]. Research observing sex-based differences in SRC incidence across individuals aged 10 years and older has demonstrated females have a higher incidence of SRC than males [31]. Finally, a review of action sports found that motocross, sailing, and snowboarding presented with the highest SRC incidence [32]. With almost a decade of literature since the Pfister et al. [30] search date, and an acceleration in SRC research during this time, there is a need for an updated review that considers all published research across youth sports.

Given the risk of SRC in young athletes, an updated understanding and awareness of the incidence of the injury may assist physicians and researchers in developing primary prevention initiatives to reduce the risk of SRC in youth athletes. Additionally, this research would be able to assist public health initiatives, coaches, parents, and the wider community in acknowledging the level of risk of SRC, so that adequate measures can be put in place to reduce this risk of SRC in young athletes. Importantly, with an updated review, it may be possible to gain a sound understanding of differences in SRC incidence between sports, ages, sex, and other important factors.

The primary aim of this systematic review and meta-analysis is to investigate the incidence of SRC in children and adolescents (≤ 18-years-old), and to assess how this differs across different sport types, age, and year of data collection. A secondary aim is to consider the impact of other potential moderating factors on SRC incidence rates, such as sex, country, setting, and level of contact.

## Methods

This systematic review and meta-analysis was registered on OSF Registries (https://osf.io/v298s). The methodology was designed and reported in accordance with the Preferred Reporting Items for Systematic Reviews and Meta-Analyses (PRISMA) statement [33] and Implementing PRISMA in Exercise, Rehabilitation, Sport medicine and Sports science (PERSiST) guidance [34].

### Equality, Diversity, and Inclusion Statement

The authorship team consisted of seven indigenous and non-indigenous authors, including three women and four men. The authorship team consisted of both junior and senior researchers, encompassing multiple disciplinary backgrounds including exercise and sports science, psychology, and nutrition.

### Search Strategy

A systematic search of electronic databases (Medline, Embase, SPORTDiscus, PsycINFO, and Web of Science) was completed without language restrictions up to September 17, 2024. No restriction was placed on year of publication. Databases were searched with relevant terms grouped by four themes. Theme 1 included terms to identify concussion outcomes (i.e., concussion incidence), theme 2 included terms to identify sports, theme 3 included terms to identify relevant populations (e.g., children and youth), and theme 4 included terms to identify relevant study designs (e.g., prospective studies and studies reporting incidence rates). For each theme, terms were searched as title words, abstract words, or database-specific subject headings where available, and the four themes were combined with the Boolean operator ‘AND’. Full search strategies for each database are provided in Supplementary Material A. Additionally, articles that were previously identified [30] that were not captured within this electronic database search process were also included.

### Eligibility Criteria

For an article to be included in the systematic review and meta-analysis it must: (i) report SRC incidence data, (ii) be a prospective cohort study, and (iii) include a sample aged ≤ 18 years. A strict definition of concussion was not used for this review as the definition of concussion has changed throughout the years. Exclusion criteria included: (i) articles that reported concussion prevalence rather than concussion incidence, (ii) studies that only reported data relating to chronic traumatic brain injury, and (iii) articles that were not published in peer reviewed journals (e.g., theses). Studies with a participant age range greater than 18 years were excluded if the mean age was greater than 18 years. Conference abstracts were also excluded due to difficulty obtaining full methods and complete data sets. During the screening process, the decision was made to exclude action sports (e.g., motocross, skiing; see Feletti and Bonato. [32]). This decision was made to provide a comparable replication of the Pfister et al. review [30].

### Selection Process and Data Extraction

Database results were exported to an external citation manager (Covidence, Veritas Health Innovation, Melbourne, Australia), that automatically identified and removed duplicates. A single reviewer then screened the title and abstract of studies based on inclusion and exclusion criteria. Following this, a full-text review was performed independently by two reviewers to assess eligibility for inclusion. Disagreements were resolved by discussion until consensus was reached. A single reviewer extracted data from all studies that fulfilled the inclusion criteria and a team of two independent reviewers cross-validated the data extracted by the initial reviewer. Any discrepancies were resolved via discussion.

The following data were extracted: number of participants, mean age, age range, sport analysed, year range of data collection, sex, level of athlete, country, setting (e.g., practice and/or competition), number of concussions, denominator (e.g., number of athlete exposures (AE) or player hours (PH)), the incidence rate (IR) of concussion, and the paper's main findings. An AE comprises of one athlete participating in one session of competition/practice during which the athlete is exposed to the possibility of athletic injury [35]. A PH is one athlete participating in one hour of sport [36]. Additional data extraction included noting whether body checking was permitted or prohibited if applicable to the sport (i.e., ice hockey), determining whether exposure values (i.e., AE or PH) were estimated or recorded as the exact value, and level of contact. Level of contact was grouped as collision, contact, combat, and non-contact determined by various studies [37–39]. When data reporting was unclear, attempts were made to contact authors for clarification.

### Quality Assessment

Following similar previous reviews [30–32], an adapted version of The Newcastle Ottawa Scale [40] was used to evaluate study quality. This component approach evaluated quality of the included studies by assessing how study cohorts were selected and concussion exposure was measured (e.g., AE or PH), whether study results were stratified by important factors in their analysis (i.e., age and/or sex), who ascertained concussion outcomes (e.g., trainer, doctor), whether concussion was defined, the duration of follow-up (e.g., number of seasons), whether concussion was measured during practice and/or games, whether mechanism of injury was reported, and whether history of concussion was reported. The study quality was assessed by a team of five reviewers that ensured that each article was independently assessed by two reviewers. These independent reviews of quality assessment data were then merged and any discrepancies in the extracted data were resolved through discussion.

### Data Synthesis and Analyses

Meta-analysis was limited to studies reporting concussion IR using AE or PH, with analyses conducted separately for each measure of incidence. Included studies that did not report AE or PH data were synthesized qualitatively by summarizing key findings and trends across these studies. Conversion of IR took place to ensure a common metric was used. AE were converted to number of concussions per 1000 exposures, while PH were converted to number of concussions per 1000 h. Given that individual studies often report multiple outcomes (e.g., data from multiple age groups, sports, or male and female participants), three-level inverse variance random-effects meta-analyses were used to account for non-independence of outcomes [41, 42]. An overall IR was calculated across all sports, with subgroup analysis conducted according to sport. Separate forest plots were generated to visualise the incidence rates (i.e., AE and PH) and 95% confidence intervals (CI) for all sports with two or more outcomes. Heterogeneity was assessed using Q, tau^2^, and *I*^2^ statistics, whereby an *I*^2^ less than 25% indicates low heterogeneity, between 25 and 50% indicates moderate, and over 50% indicates high heterogeneity [43].

When data were available, meta-regression analyses were conducted (separately for AE and PH) according to the year of data collection and participant mean age. When data were collected over multiple years or reported as an age range, the median of this period was used for analysis. To ensure inclusion of a single Canadian Football study, it was grouped with American Football for the meta-analysis. Given that taekwondo had substantially higher IR compared to all other sports, it was excluded from the meta-regression analyses. Results were visualized with bubble plots, coloured by level of contact, although level of contact was not included in the meta-regression models. When data were available for potential moderator analyses these were generated, which included sex, country, setting (practice or competition), exposure measurements (estimated or exact), body checking (permitted or prohibited), level of contact, and whether studies reported their sample size. Finally, given that sex and sport have been moderators of concussion IR in previous studies, moderator analyses according to sex within sports were conducted. Outliers were expected as data were pooled across a diverse range of sports. All analyses were performed using the statistical program R version 4.2.2 (R Foundation for Statistical Computing, Vienna, Austria) and the *tidyverse* [44], *metafor* [45], *meta* [46], *flextable* [47] packages.

## Results

### Identification of Studies

**Fig. 1:**
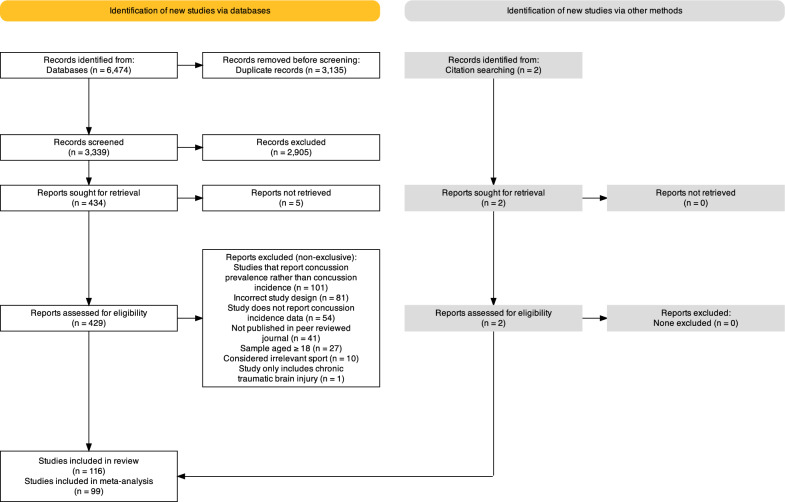
Flowchart of the selection process for inclusion of articles in the systematic review and meta-analysis

Figure 1 reports the PRISMA flowchart representing the process that led to the studies included in the systematic review and meta-analysis. The initial search yielded 6474 citations, whereby 3135 duplicates were removed. Of the remaining 3339 citations, 2905 were excluded after screening by title and abstract. There were 434 articles that met criteria for a full text review; however, five articles were unable to be retrieved. Two additional studies were identified through the findings of the previous review [30]. Following the exclusion of 315 citations, 116 studies were included in the systematic review, 99 of which were included in the meta-analysis.

### Details of Included Studies

Table 1 displays the characteristics of studies included in both the systematic review and meta-analysis, and Table 2 displays the characteristics of studies only included in the systematic review. A total of 3,025,911 participants were included; however, 40.5% of studies did not report sample size. Studies that reported sample size by sex included a total of 1,591,024 (58.7%) males and 1,119,208 (41.3%) females. The publication dates ranged from 1992 to 2024 and 28 sports were identified. These included American Football (n = 40), ice hockey (n = 25), lacrosse (n = 23), soccer (n = 24), basketball (n = 22), rugby union (n = 19), wrestling (n = 16), volleyball (n = 16), softball (n = 15), baseball (n = 15), cheerleading (n = 11), field hockey (n = 6), track and field (n = 6), swimming and diving (n = 5), cross country (n = 4), taekwondo (n = 3), Australian Rules football (n = 3), rugby league (n = 2), gymnastics (n = 3), tennis (n = 2), golf (n = 2), water polo (n = 2) and one study each for crew/rowing, rugby 7s, dance, martial arts, bowling, and boxing. There were 75 studies that were based in the USA, 18 in Canada, nine in Australia, four in England, three in Ireland, two in South Africa, and one each in Greece, New Zealand, Japan, and South Korea. There was one study that did not specify a country. Among the 116 studies, 61 reported AE as the denominator for IR calculations, 38 reported PH, seven studies reported by player years, four studies reported by number of players, three reported by player seasons, two reported by number of games, and one study reported by number of athlete days per year.

**Table 1:** Details of studies included in the systematic review and meta-analysis

Study	Country	Sports	Sex	Sample size (males/females)	Age information	Exposure measure	Main findings
Archbold et al. [48]	Ireland	Rugby Union	Male	825 (825/0)	Age range (SD) = 16.8 (0.8)	1000 PH	Sprain, concussion, and muscle injury were the most common diagnoses, and concussion carried the most significant time out from play
Barden et al. [49]	England	American Football, Basketball, Soccer, Rugby Union, and Rugby League	Male, Female	843 (NR/NR)	Mean age (SD) = 17.7 (1), Age range (16–20)	1000 PH	Concussion was the most common injury and female rugby union had the highest concussion incidence (50%)
Baron et al. [50]	USA	Lacrosse	Female	NR (0/NR)	NR	1000 AE	The player wearing headgear demonstrated significant decreases in game concussion when compared to players not wearing headgear
Beis et al. [51]	Greece	Taekwondo	Male, Female	1990 (1223/767)	Age range (11–17)	1000 AE	Junior boys and girls (aged 11 to 13 years) sustained more concussions than older age groups
Black et al. [52]	Canada	Ice Hockey	Male, Female	1331 (1273/43)^a^	Age range (11–12)	1000 PH	Pee Wee hockey players that played in a body checking permitting league had a threefold greater rate of concussion compared with a league where body checking was prohibited due to policy change
Blake et al. [53]	Canada	Ice Hockey	Male	1208 (1208/0)	Age range (11–17)	1000 PH	There was a higher concussion rate in ice hockey players who did not meet the Physical Activity recommendation guidelines in comparison to players that did meet these recommendations
Clifton et al. [54]	USA	Basketball	Female	NR (0/NR)	NR	1000 AE	The most common injuries for basketball female athletes were ligament sprains, concussions, and muscle/tendon strains
Clifton et al. [55]	USA	Basketball	Male	NR (NR/0)	NR	1000 AE	The most common injuries for basketball male athletes were ligament sprains, concussions, and muscle/tendon strains
Collins et al. [56]	USA	American Football	Male	NR (NR/0)	NR	10,000 AE	Concussion rates significantly increased from 2008–2009 through 2012–2013
Cosgrove et al. [57]	Ireland	Rugby Union	Male	135 (135/0)	Mean age (SD) = 16.7 (0.8), Age range (15–19)	1000 PH	Match concussion incidence was higher than training concussion incidence
DiStefano et al. [58]	USA	Soccer	Female	NR (0/NR)	NR	1000 AE	Concussions accounted for 24.5% of competition injuries in high school soccer
Dompier et al. [59]	USA	American Football	NR	NR (NR/NR)	Age range (5–23)	1000 AE	Game concussion IR was higher than in practice IR across youth, high school, and college American Football athletes
Echlin et al. [60]	Canada	Ice Hockey	Male	67 (67/0)	Mean age (SD) = 18.2 (1.2), Age range (16- 21)	1000 AE	The IR of concussions during junior ice hockey games was seven times higher than the highest rate reported in previous studies
Eliason et al. [61]	Canada	Ice Hockey	Male, Female	1647 (NR/NR)	Age range (13–14)	1000 PH	Among ice hockey players aged 12–14 years in leagues permitting body checking, there was no significant difference in concussion IR between athletes with and without prior body checking experience
Eliason et al. [62]	Canada	Ice Hockey	Male, Female	941 (NR/NR)	Age range (15–17)	1000 PH	Players with three or more years of body checking experience had higher concussion rates when compared to players with two years or less experience
Eliason et al. [63]	Canada	Ice Hockey	Male, Female	1344 (NR/NR)	Age range (11–17)	1000 PH	Players participating in lower levels of play, and those with an injury or concussion history, had higher rates of concussion. Goalkeepers and players in leagues that disallowed bodychecking had lower rates
Emery et al. [64]	Canada	Ice Hockey	Male, Female	1971 (1956/15)	Age range (11–14)	1000 PH	The risk of concussion was reduced for ice hockey players (aged 13–14 years) who had two years of bodychecking experience previously compared with players introduced to bodychecking for the first time at age 13
Emery et al. [65]	Canada	Ice Hockey	Male, Female	1004 (987/17)	Age range (13–14)	1000 PH	Whilst there was a lower IR of concussion within ice hockey leagues prohibiting body checking compared to leagues permitting, it was not statistically significant
Emery et al. [66]	Canada	Ice Hockey	Male, Female	1127 (1118/8)^a^	Age range (15–17)	1000 PH	The rate of concussion injury was 51% lower in ice hockey leagues not permitting body checking in for non-elite 15–17-year-olds
Emery et al. [67]	Canada	Ice Hockey	Male, Female	2154 (2117/33)^a^	Age range (11–12)	1000 PH	In ice hockey teams where body checking was permitted, there was a threefold increase in concussions, compared to leagues that prohibited body checking
Emery and Meeuwisse [68]	Canada	Ice Hockey	Male, Female	986 (962/24)	Age range (8–17)	1000 PH	Concussions were the most common specific injury type for ice hockey players, followed by shoulder sprains/dislocation and knee sprains
Fremont et al. [69]	Canada	Canadian Football^b^	Male	672 (672/0)	Age range (11–17)	1000 AE	The concussion IR was over six times higher than the highest incidence rate reported for high school football between 1999 and 2012
Gessel et al. [70]	USA	American Football, Basketball, Volleyball, Soccer/Football, Wrestling, Baseball, and Softball	Male, Female	NR (NR/NR)	NR	1000 AE	Rates of concussion were highest in football and soccer, and high school girls sustained a higher rate of concussions than boys
Gomez et al. [71]	USA	Basketball	Female	890 (0/890)	Age range (14–18)	1000 PH	Of the injuries that occurred in basketball, concussion accounted for 2%
Guillaume et al. [72]	USA	Lacrosse	Male	NR (NR/0)	NR	1000 AE	When rule modifications were in effect, concussion and overall injury risks decreased for both players (body checker and player being body checked)
Guskiewicz et al. [73]	USA	American Football	NR	17,549 (NR/NR)	NR	1000 AE	The greatest incidence of concussion occurred at the high school level and collegiate division III level for American Football athletes, suggesting that there was no association between levels of play and the proportion of injury
Hancock et al. [74]	England	Rugby Union	Male	NR (NR/0)	Age range (11–18)	1000 PH	Injury incidence and burden were higher in U18 than U13 and U15 age groups
Haseler et al. [75]	England	Rugby Union	Male	210 (210/0)	Age maximum (< 17)	1000 PH	Concussion affected half of the total head injuries that occurred for the rugby union players
Hecimovich and King [76]	Australia	Australian Rules Football	Male	976 (976/0)	Age range (9–17)	1000 AE	Australian Rules football players aged 14 -17 had the highest rates of concussion in comparison to younger players
Herman et al. [77]	USA	Lacrosse	Female	NR (0/NR)	NR	1000 AE	The concussion injury rate was higher in the no headgear mandate in comparison to the headgear mandated lacrosse athletes
Hinton et al. [78]	USA	Lacrosse	Male, Female	NR (NR/NR)	Age range (15 -18)	1000 AE	Male lacrosse players had higher rates of concussion from player-to-player contact in comparison to females
Junge et al. [79]	New Zealand	Soccer and Rugby Union	Male	268 (268/0)	Mean age (SD) = 16.7 (.97), Age range (14–18)	1000 PH	More concussions occurred in the rugby union players in comparison to the soccer players
Kawasaki et al. [80]	Japan	Rugby Union	Male	600 (600/0)	Mean age (SD) = 17.4 (0.7)	1000 PH	U18 athletes had a lower incidence rate than U22 and elite athletes
Kerr et al. [81]	USA	Lacrosse	Male	550 (550/0)	Mean age (SD) = 12 (2), Age range (9–15)	1000 AE	Concussion injuries for these lacrosse players were higher in games than practices overall
Kerr et al. [82]	USA	American Football, Ice Hockey, Lacrosse, Wrestling, Soccer, Basketball, Baseball Swimming, Track and Field, Cross Country, Volleyball, Field Hockey, and Softball	Male, Female	NR (NR/NR)	NR	10,000 AE	Competition had a higher concussion compared to practice, and boys’ football had the highest concussion rate, followed by girls’ soccer and boys’ ice hockey
Kerr et al. [83]	USA	American Football	Male	664 (664/0)	Mean age (SD) = 12 (1), Age range (10–14)	1000 AE	The proportion of injuries diagnosed as concussions was higher in competition than practice for these American Football youth athletes
Kerr et al. [84]	USA	Baseball, Basketball, American Football, Soccer, Track, Wrestling, Cheerleading, Softball, and Volleyball	Male, Female	NR (NR/NR)	NR	1000 AE	Football had the highest concussion rate and concussion rates were higher in games versus practices
Kerr et al. [85]	USA	American Football	NR	390 (NR/NR)	NR	1000 AE	The concussion rate was lower in high schools where player safety coach intervention had been implemented, in comparison to players that received education only
Kerr et al. [86]	USA	Volleyball	Female	NR (0/NR)	NR	1000 AE	Volleyball players playing in the libero position had a high incidence of concussion
Kerr et al. [87]	USA	American Football	NR	NR (NR/NR)	Mean age (SD) = 10.7 (1.9), Age range (5–14.9)	1000 AE	The most common injuries included contusions, ligament sprains, concussions, and muscle strains for these American Football youth athletes
Kerr et al. [88]	USA	Ice Hockey	Male	NR (NR/0)	Age range (14–18)	1000 AE	The concussion rate was higher in competition in comparison to practice, and most concussions occurred after the first period of ice hockey games
Kerr et al. [89]	USA	Soccer	Male	NR (NR/0)	NR	1000 AE	Concussions accounted for more than one fifth of injuries in high school soccer games
Kerr et al. [90]	USA	Lacrosse	Male	NR (NR/0)	NR	1000 AE	The concussion rate in youth lacrosse players was higher than those in high school and college
Kerr et al. [91]	USA	American Football	Male	NR (NR/0)	NR	1000 AE	Concussions were a common injury during competitions amongst most positions for American Football youth athletes
Kerr et al. [92]	USA	American Football	NR	2108 (NR/NR)	Mean age (SD) = 10.88 (1.92), Age range (5.2–15.64)	1000 AE	Most concussions were reported in the group of American Football youth athletes that received no Heads-Up Football Program compared to those that did receive this education program. However, no statistical difference in concussion rates were found between groups
Kerr et al. [93]	USA	American Football	NR	2098 (NR/NR)	Age range (5–15)	1000 AE	Concussion injury rates were greater in American Football players aged 11 to 15 years old in comparison to five to 10 years old
Kerr et al. [94]	USA	American Football	NR	NR (NR/NR)	Youth age range (5–14)	1000 AE	Concussions in high school football had the highest mean number of reported symptoms, followed by college and youth
Koh and Cassidy [95]	South Korea	Taekwondo	Male, Female	2328 (1652/676)	Mean age (SD) = 15 (1.62), Age range (11–19)	1000 AE	The incidence of concussions was high in competition taekwondo
Kolstad et al. [96]	Canada	Ice Hockey	Male, Female	3330 (NR/NR)	Age range (11–18)	1000 PH	Wearing a mouthguard was associated with a lower concussion rate for youth ice hockey players
Kontos et al. [97]	USA	American Football	Male	468 (468/0)	Mean age (SD) = 10.12 (1.31), Age range (8–12)	1000 AE	Participation in American Football games was associated with an increased concussion risk compared to practices, and players aged 11–12 years were nearly three times more likely to suffer a concussion compared to younger players
Kontos et al. [98]	USA	Ice Hockey	Male, Female	397 (330/67)	Mean age (SD) = 14.73 (1.99), Age range (12–18)	1000 AE	The concussion incidence rate was higher during ice hockey games compared to practices
Kroshus et al. [99]	USA	Wrestling	Male	NR (NR/0)	NR	1000 AE	For high school male wrestlers, the most common occurring injuries during competitions were concussions, followed by knee and ankle sprains
Leahy et al. [100]	Ireland	Rugby Union	Male	NR (NR/0)	Age range (16–19)	1000 PH	Concussions carried the highest injury burden for rugby union forward position players, compared to other positions
Leung et al. [101]	Australia	Rugby Union	Male	3585 (3585/0)	Age range (9–18)	1000 PH	The incidence of suspected concussion injuries was 4.3/1,000 PH, and injuries differed across age groups
Leung et al. [102]	Australia	Rugby Union	Male	480 (480/0)	Age range (10–18)	1000 PH	Concussions accounted for 19% of all injuries reported for these rugby union youth athletes
Lincoln et al. [103]	USA	American Football, Lacrosse, Soccer, Wrestling, Basketball, Baseball, Softball, Field Hockey, and Cheerleading	Male, Female	158,430 (NR/NR)	NR	1000 AE	American Football had the highest incidence rates, whilst girls’ soccer had the most concussions amongst the sports participated by girls
Lincoln et al. [104]	USA	Lacrosse	Male, Female	8638 (5072/3566)	NR	1000 AE	Concussion was the most common injury and male lacrosse players had a higher percentage of concussions in comparison to females
Lincoln et al., (2014) [105]	USA	Lacrosse	Male, Female	NR (NR/NR)	Age range (9–15)	1000 AE	Whilst there were four concussions amongst the male lacrosse players, no concussions were reported amongst the females
Lopez et al. [106]	USA	Rugby 7 s	Male, Female	3804 (3072/732)	Age maximum (< 19)	1000 PH	The most common overall injury types were ligament sprains, concussions, and contusions
Lynall et al. [107]	USA	Field Hockey	Female	NR (0/NR)	NR	1000 AE	Concussions accounted for almost 25% of all competition injuries among high school field hockey players
Lynall et al. [108]	USA	Ice Hockey	Male	NR (NR/0)	NR	1000 AE	In both practice and competition, the majority of injuries occurred in the head/face and shoulder/clavicle and resulted in concussions, contusions, or ligament sprains
Makovec Knight et al. [109]	Australia	Australian Rules Football	Male, Female	400 (230/170)	Mean age (SD) = 10.6 (1.2), Age range (7–13)	1000 PH	Head gear use in Australian Rules Football youth players was not found to be associated with suspected concussions
Marshall et al. [110]	USA	American Football, Soccer, and Lacrosse	Male, Female	7513 (NR NR)	NR	100,000 AE	Football had the highest concussion IR, followed by women’s lacrosse, men’s lacrosse, men’s soccer, and women’s soccer
Marshall and Spencer [111]	USA	Rugby Union	NR	NR (NR/NR)	NR	1000 AE	Games had a higher concussion rate than practices and concussions were responsible for 25% of all days lost in rugby participation due to injury
McFie et al. [112]	South Africa	Rugby Union	NR	7216 (NR/NR)	Under 13 Median age = 13.3, Under 16 Median Age = 16.3, Under 18 Median Age = 17.8, Age maximum (< 18)	1000 PH	Under 13 players and Under 16 players had higher concussion incidence rates than Under 18 players
McGinnis et al. [113]	USA	Lacrosse	Male	NR (NR/0)	Mean age (SD) = 14.8 (2.12), Age range (8–18)	1000 AE	The most common injury diagnoses for male lacrosse players included contusions, concussions, fractures and sprains
McGuine et al. [114]	USA	American Football	NR	2081 (NR/NR)	Mean age (SD) = 15.9 (1.2)	1000 AE	The SRC rate in competition was significantly higher than in practice for these American Football youth athletes
McIntosh and McCrory [115]	Australia	Rugby Union	Male	294 (294/0)	Age maximum (< 15)	1000 AE	The was no significant difference in injury rates (including concussion) between rugby union players with and without headgear
McIntosh et al. [116]	Australia	Rugby Union	Male	4095 (4095/0)	Age range (12–21)	1000 PH	The results of this study suggested that padded headgear does not reduce the rate of head injury or concussion for these rugby union players as no differences were found in the concussion rate between control groups and headgear groups
McMahon et al. [117]	Australia	Australian Rules Football	Male, Female	1253 (1236/17)	Age maximum (< 15)	1000 PH	Three out 15 cases of concussions resulted in a loss of consciousness and there were more concussions recorded in the older age group (Under 15) in comparison to the younger age groups
Messina et al. [118]	USA	Basketball	Male, Female	1863 (973/890)	Age range (14–18)	1000 PH	There was a reported higher number of concussions in boy compared to girl basketball players
Murray-Smith et al. [119]	Australia	Rugby Union	Male	979 (979/0)	Age maximum (< 17)	1000 PH	Concussion accounted for the greatest injury burden out of all injuries diagnosed for these rugby union players
O’Connor et al. [120]	USA	American Football, Wrestling, Field Hockey, Gymnastics, Volleyball, Baseball, Softball, Basketball, Crew/Rowing, Cross Country, Golf, Lacrosse, Soccer, Indoor Track and Field, Outdoor Track and Field, Swimming and Diving, and Tennis	Male, Female	NR (NR/NR)	NR	10,000 AE	Football has the highest SRC rate, followed by boys’ lacrosse and girls’ soccer. The SRC rate was higher in competition in comparison to practice
O’Kane et al. [121]	USA	Soccer	Female	351 (0/351)	Age range (11–14)	1000 AE	The concussion rate in games was greater than that in practices, the Under 15 female soccer players had the highest rate of concussion, and the Under 14 players had the lowest rate of concussion
Peek et al. [122]	Australia	Soccer	Male, Female	364 (226/138)	Age range (12–18)	1000 PH	Neuromuscular neck exercise group reported fewer concussions than the comparison group
Peterson et al. [123]	USA	American Football	Male, Female	3,794 (NR/NR)	Age range (8–13)	1000 AE	Concussions were more frequent during game than practice for the American Football youth players. Players in the sixth or seventh grade had a higher likelihood of experiencing concussions in comparison to younger players
Pfaller et al. [124]	USA	American Football	NR	NR (NR/NR)	Mean age (SD) = 15.9 (1.2)	1000 AE	The concussion rate overall in American Football practice was significantly lower after the rule change in 2014 that limited the amount and duration of full contact
Pierpoint et al. [125]	USA	Lacrosse	Female	NR (0/NR)	NR	1000 AE	Concussion was the most common diagnosis among all high school female lacrosse player positions
Pierpoint et al. [126]	USA	Lacrosse	Male	NR (NR/0)	NR	1000 AE	Concussion was the most frequent competition diagnosis for all high school male lacrosse player positions
Pieter and Zemper [127]	USA	Taekwondo	Male, Female	4,258 (3,341/917)	Age range (6–16)	1000 AE	Contusions were the most common injury occurring for boys and girls, followed by concussion
Powell and Barber-Foss [128]	USA	Baseball, Basketball, American Football, Soccer, Wrestling, Field Hockey, Softball, and Volleyball	Male, Female	NR (NR/NR)	NR	1000 AE	Of the mild traumatic brain injuries, American Football accounted for 63.4% of them, followed by wrestling (10.5%) and girls’ soccer (6.2%)
Rivara et al. [129]	USA	American Football, Soccer	Male, Female	778 (490/288)	Age range (14–19)	1000 AE	The cumulative incidence of concussions was more than 10% for both high school football and girls’ soccer players
Roberts et al. [162]	USA	Ice Hockey	Male, Female	807 (695/112)	Age range (11–19)	1000 PH	Male ice hockey players had a higher rate of concussion at all age levels in comparison to female players
Schneider et al. [130]	Canada	Ice Hockey	Male, Female	778 (659/119)	Age range (13–17)	1000 PH	The rate of concussion in this ice hockey study was found to be higher than previously reported from the same league and the rate of concussion in males and females were not significantly different
Schulz et al. [131]	USA	American Football, Soccer, Wrestling, Basketball, Softball, Baseball, Track, and Cheerleading	Male, Female	15,802 (NR/NR)	NR	100,000 AE	Concussion rates were elevated for athletes with a history of concussion, and they increased with the increasing level of body contact allowed in the sport. American Football had the overall highest concussion rate
Sewry et al. [132]	South Africa	Rugby Union	NR	NR (NR/NR)	NR	1000 PH	The trend in concussions for rugby union players decreased until 2013 and increased in 2014 – 2016, consistent with the overall injury rate trend
Shill et al. [133]	Canada	Rugby Union	Female	421 (0/421)	Age range (15–18)	1000 PH	The concussion rate decreased from 2018 and 2019 for rugby union female players and tackling was the most frequent mechanism of concussion in matches
Shill et al. [134]	Canada	Rugby Union	Male, Female	902 (481/421)	Age range (15–18)	1000 PH	The rate of concussion was significantly higher in females than males in the Canadian high school cohort
Smith et al. [135]	USA	American Football, Soccer, Basketball, Wrestling, Baseball, Volleyball, Softball, Ice Hockey, Lacrosse, Swimming and Diving, Cheerleading, and Track and Field	Male, Female	NR (NR/NR)	NR	10,000 AE	High school sports players located in an environment with a higher altitude demonstrated a 31% reduction in the incidence of total reported concussions
Tee et al. [136]	England	Rugby League	NR	81 (NR/NR)	Mean age (SD) = 17.8 (0.7)	1000 PH	Concussion and ankle sprains were the most common injuries for these rugby league players
Tuominen et al. [137]	NR	Ice Hockey	Male	NR (NR/0)	Age maximum (< 18)	1000 PH	Concussion was the most common head and face injury in the Ice Hockey World Championship Under-18 tournament
Vaandering et al. [138]	Canada	Volleyball	Male, Female	1,876 (466/1,391)^a^	Mean age (SD) = 16.2 (1.26), Age maximum (< 18)	1000 AE	Joint sprains and concussions were the most common injuries for these volleyball athletes
Valier et al. [139]	USA	Softball	Female	NR (0/NR)	NR	1000 AE	The most frequent time loss injuries for female softball players were concussions, strains and sprains
Warner et al. [167]	USA	Lacrosse	Male, Female	NR (NR/NR)	Male mean age (SD) = 16.2 (1.3), Female mean age (SD) = 16 (1.2)	10,000 AE	The most frequently diagnosed injury for both male and female lacrosse players were concussions
Wasserman et al. [140]	USA	Softball	Female	NR (0/NR)	NR	1000 AE	Softball players sustained a variety of injuries, with the most being ankle sprains and concussions
West et al. [141]	Canada	Rugby Union	Male	429 (429/0)	NR	1000 PH	The rate of injury and concussion in Canadian youth high school male rugby was high, with tackle-related injuries and concussion being the most common
Williams et al. [142]	USA	Volleyball	Female	NR (0/NR)	NR	1000 AE	The most diagnoses reported for these female volleyball players included sprains, concussions and contusions
Yard and Comstock [143]	USA	American Football, Soccer, Basketball, Wrestling, Baseball, Volleyball, and Softball	Male, Female	NR (NR/NR)	NR	100,000 AE	Concussion rates were highest in football, girls’ soccer, boys’ soccer, girls’ basketball and wrestling. Concussion rates were higher in competition in comparison to practice
Zemper [144]	USA	American Football	Male	NR (NR/0)	NR	1000 AE	American Football youth athletes with a history of concussion were six times more likely to sustain a concussion compared to athletes with no history of concussion

AE, Athlete Exposures; PH, Player Hour; USA, United States of America; NR, No Response

^a^Study included participants with missing sex data

^b^Canadian Football was classified as American Football for the purposes of the meta-analysis

**Table 2:** Details of studies included in the systematic review only

Study	Country	Sports	Sex	Sample size (males/females)	Age information	Exposure measure	Main findings
Ali et al. [145]	USA	American Football, Soccer, Basketball, Lacrosse, Gymnastics, Cheerleading, Wrestling, Boxing, Martial Arts, Water Polo, Diving, Baseball, Softball, Volleyball, and Track and Field	Male, Female	7453 (NR/NR)	Mean age = 15.4, Age range (12–22)	Per 100 patient years	The incidence of concussion among athletes with chronic headaches was 55.6 concussions per 100 patient years, whilst future concussion incidence was lower among athletes who did not have chronic headaches at 43.0 concussions per 100 patient years
Ali et al. [146]	USA	American Football, Soccer, Basketball, Volleyball, Lacrosse, Baseball, Softball, Cheerleading, and Wrestling	Male, Female	7453 (NR/NR)	Mean age = 15.4, Age range (12–22)	Per 100 patient years	No difference in concussion incidence was found between the unmedicated athletes with anxiety/depression (50.2 concussions per 100 patient years) and the group without anxiety/depression or antidepressant use (52.6 concussions per 100 patient years). However, athletes with anxiety and depression taking antidepressants had a significantly greater incidence of concussion (89.7 concussions per patient years) compared to both other groups
Ali et al. [147]	USA	American Football, Lacrosse, Wrestling, Ice Hockey, Soccer, Basketball, Volleyball, Baseball, Softball, and Cheerleading	Male, Female	7453 (NR/NR)	Age range (12–22)	Per 100 patient years	Athletes with ADHD and using stimulant medication experienced fewer concussions (37.3 concussions per 100 patient years) than those athletes with ADHD not using medication (57.0 concussions per 100 patient years) and non-ADHD athletes (52.8 concussions per 100 patient years)
Bretzin et al. [160]	USA	Basketball, Baseball, American Football, Ice Hockey, Lacrosse, Soccer, Swimming and Diving, Wrestling, Cheerleading, Softball, and Volleyball	Male, Female	193,757 (116,434/77,323)	NR	Per 100 player seasons	The overall clinical incidence for all sports was 1.7 per 100 player seasons, 1.9 per 100 player seasons for male sports and 1.5 per 100 player seasons for female sports. Females were at a 1.9 times greater risk of a concussion than male athletics in sex-comparable sports
Bretzin et al. [161]	USA	Soccer	Male, Female	83,378 (43,741/39,637)	NR	Per 100 athlete seasons	The overall incidence of concussion was 1.8 per 100 athletes per season. Female soccer players had a higher risk of concussion than their male counterparts
Bretzin et al. [156]	USA	American Football, Basketball, Soccer, Wrestling, Volleyball, Cheerleading, Lacrosse, Softball, Ice Hockey, Baseball, Swimming and Diving, Track and Field, Gymnastics, Tennis, Cross-country, Golf, Water Polo, Field Hockey, Bowling	Male, Female	2,182,128 (1,267,389/914,739)	NR	Per 100 player seasons	The overall clinical incidence of concussion for all sports was 1.17 per 100 player seasons, 1.34 per 100 player seasons for male sports and 0.93 per 100 player seasons for female sports. Girls had higher incidence than boys in softball/baseball, basketball, and soccer
Covassin et al. [154]	USA	American Football, Ice Hockey, Soccer, Basketball, Wrestling, Cheerleading, Lacrosse, Basketball, Volleyball, Softball, and Baseball	Male, Female	182,719 (110,774/71,945)	NR	Per 100 athletes	The overall incidence of concussion across all sports was 2.36 per 100 student athletes. The highest concussion incidence was recorded in American Football, women’s basketball and women’s soccer
DeLee and Farney [148]	USA	American Football	NR	4399 (NR/NR)	NR	Per 1000 players	The overall concussions for American Football high school players in Texas within this study was 101 concussions amongst 4399 players (22.9 of 1,000 players)
Dugan et al. [155]	USA	Basketball, Cheerleading, American Football, Ice Hockey, Soccer, Volleyball, Gymnastics, Lacrosse, Baseball, Dance, Cross Country, and Wrestling	Male, Female	67,212 (NR/NR)	NR	Per 100 athletes	American Football and boys’ and girls’ ice hockey had the highest rate of concussion (6 per 100 athletes)
Hannah et al. [149]	USA	American Football and other sports	Male, Female	11,563 (7622/3491)	Female mean age (SD) = 15.33 (1.54), Male mean age (SD) = 15.37 (1.56), Median age = 15, Age range (12–22)	Per 1000 patient years	The incidence of concussion per 1000 person years was not significantly different between male (170 per 1000 person years) and female youth athletes (185.9 per 1000 person years)
Meyers [150]	USA	American Football	NR	NR (NR/NR)	NR	Per 10 team games	There was a significantly lower rate of concussions for American Football athletes competing on heavier infill weight systems artificial turf (0.8 to 0.3 per 10 team games) compared with the lighter infill weight systems (0.4 to 0.3 per 10 team games)
Meyers and Barnhill [151]	USA	American Football	NR	NR (NR/NR)	NR	Per 10 Team Games	Greater incidence of concussion was observed during competition on natural grass (1.8 per 10 team games) when compared to competition on FieldTurf (.07 per 10 team games) for these American Football youth athletes
Morrissey et al. [157]	USA	Ice Hockey	Female	NR (0/NR)	Age maximum (< 18)	Per 10,000 athletes	The overall traumatic brain injury rate was 60.0 per 10,000 athletes across all age groups under 18 years of age. Female ice hockey players aged 15–18 had the highest concussion injuries (150.1) in comparison to younger aged children
Spiera et al. [152]	USA	American Football and other sports	Male, Female	11,380 (NR/NR)	Mean age control cohort (SD) = 15.35 (0.02), Mean age anti-inflammatory medication cohort (SD) = 15.42 (0.09)	Per person years	No significant difference was found for concussion incidence between the athletes who did not use anti-inflammatory medication (0.18 per person years) and the athletes that use anti-inflammatory medication (0.17 per person years)
Tisano et al. [158]	USA	Gymnastics	Male, Female	NR (NR/NR)	Age range (7–17)	Per 100,000 athlete days	Concussions were more common in adolescent gymnastics athletes (1.05 per 100,000 athlete days) compared to children (0.33 per 100,000 athlete days)
Zendler et al. [153]	USA	Basketball, American Football, and Soccer	Male, Female	NR (NR/NR)	Age range (6–18)	Per 100,000 participant years	The rate of concussions in non-tackle football (40.7 per 100,000 participant years) was three times less than that in basketball (124.9 per 100,000 participants) or soccer (138.6 per 100,000 participants)
Zynda et al. [159]	USA	Basketball	Male, Female	NR (NR/NR)	Age range (7–17)	Per 100,000 participant years	The rate of concussion head injuries in female basketball athletes increased significantly from childhood (4.9 per 100,000 participants) to adolescence (19 per 100,000 participants) compared with that in male childhood (5.9 per 100,000 participants) and adolescent athletes (8.5 per 100,000 participants)

USA, United States of America; NR, No Response

### Study Quality

The quality assessment of included studies is presented in Supplementary Material B. All 116 studies appeared to have a representative exposed cohort. Only 63 studies (54%) stratified by important factors, such as age or sex. Regarding the quality of outcomes measures, 58 studies (50%) provided a definition for concussion, team trainers were tasked with reporting the number of concussions for 74 studies (64%) and mechanism of injury was provided by 63 studies (54%). There were 78 studies (67%) that recorded concussion IR across both practice and competition. There were 115 studies that reported a duration of follow-up, with the longest duration being 18 years. Participant history of concussion was only explored by 38 studies (33%).

### Qualitative Synthesis

For studies that were not included in the meta-analyses due to not reporting AE or PH, evidence suggested that American Football SRC incidence was high compared to many other sports [154–156]. Many studies tended to explore the influence of additional variables and the impact on SRC incidence, such as chronic headaches, medications, environmental altitudes, and different field turfing environments. Additionally, multiple studies investigating incidence across multiple sports reported that SRC incidence was higher in adolescents compared to children [157–159] and females had a higher risk of SRC than males [156, 160, 161]. Indeed, in a large epidemiological study spanning 2015–2023, girls consistently had higher incidence rates than boys in baseball/softball, basketball, and soccer [156].

### Meta-Analyses

#### Incidence of Concussion by Athlete Exposures

**Fig. 2:**
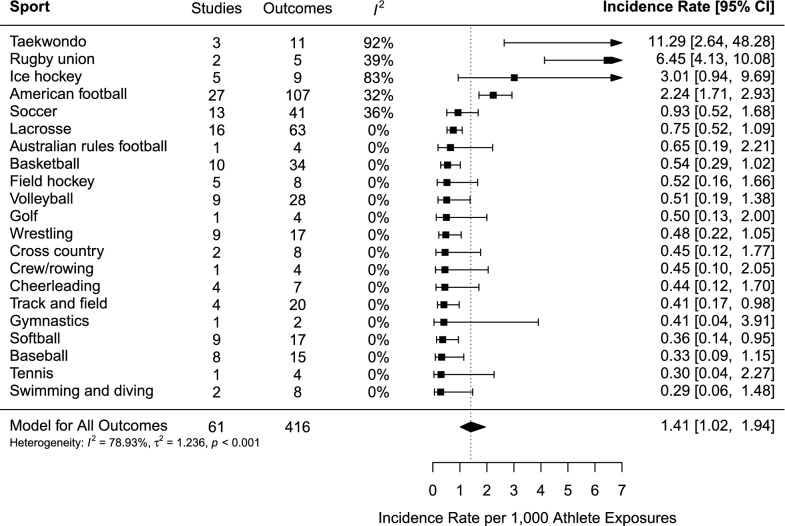
Forest plot of the sport-specific concussion incidence rates per 1000 athlete exposures. *Note*: Three variables are truncated and indicated with arrowheads to the right of the plot. The arrowheads denote that confidence intervals extend beyond the plotted range

The pooled incidence of SRC per 1000 AE across 21 sports from 61 studies was 1.41 (95% CI 1.02–1.94), with high (*I*^2^ = 79%) and significant (*p* < 0.001) heterogeneity (Fig. 2). The incidence of SRC ranged from 0.29 (95% CI 0.06–1.48) for swimming and diving to 11.29 (95% CI 2.64–48.28) for taekwondo. Whilst taekwondo reported the highest SRC incidence, it also had high heterogeneity (92%), indicating the potential existence of outliers. Apart from taekwondo, rugby union (IR = 6.45, 95% CI 4.13–10.08), ice hockey (IR = 3.01, 95% CI 0.94–9.69), and American Football (IR = 2.24, 95% CI 1.71–2.93) showed the highest incidence.

**Fig. 3:**
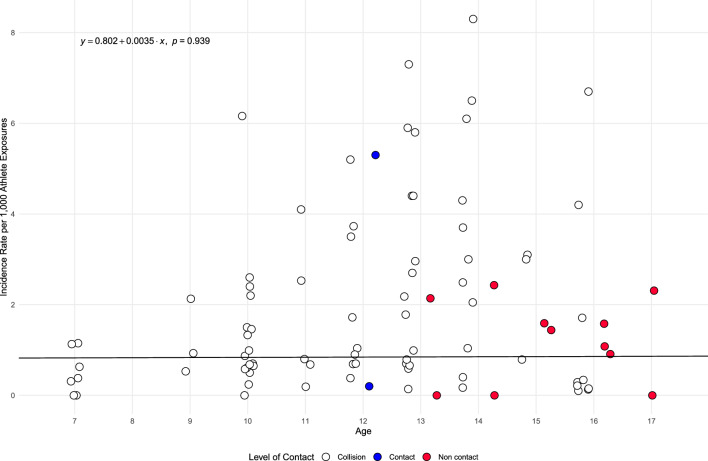
Bubble plot exploring the relationship between age and concussion incidence per 1000 athlete exposures. *Note*: Points have a jitter along the x-axis to improve readability

**Fig. 4:**
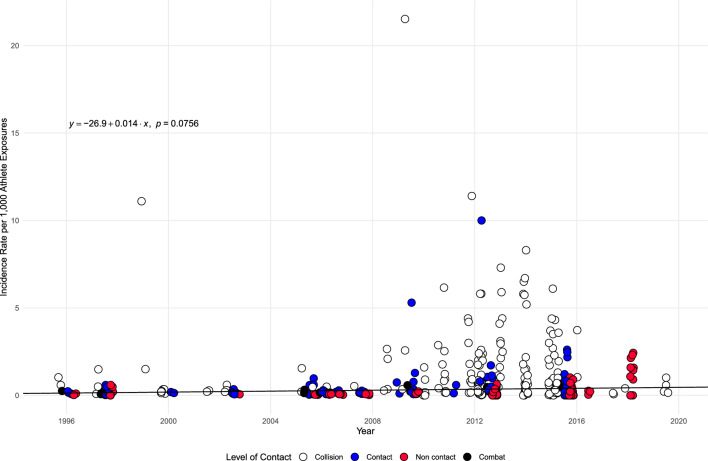
Bubble plot exploring the relationship between year and concussion incidence per 1000 athlete exposures. *Note*: Points have a jitter along the x-axis to improve readability

The meta-regression analysis examining the relationship between age and incidence of SRC per 1000 AE, as displayed in Fig. 3, was non-significant (*p* = 0.939), suggesting age did not moderate IR. The meta-regression analysis examining the relationship between the year of data collection and incidence of SRC per 1000 AE, as displayed in Fig. 4, was non-significant (*p* = 0.076), suggesting year did not moderate incidence.

As presented in Table 3, moderator analyses found that within studies that reported sex -specific incidence by AE, there was no significant difference found between males and females (*p* = 0.157) or between AE that were estimated or exact measurements (*p* = 0.207). The results revealed that there was a significant difference in SRC incidence amongst various settings (*p* < 0.001). Specifically, competition was found to have the highest incidence (IR = 2.39) compared to practice (IR = 0.51) and practice and competition settings (IR = 0.47). Level of contact was found to be a significant moderator of SRC incidence (*p* < 0.001), whereby collision sports had the highest incidence (IR = 1.72). Country was found to be a significant moderator of SRC incidence (*p* < 0.001), with South Korea reporting the highest incidence (IR = 47.12). However, it is worth noting that incidence within South Korea was represented by one study that explored taekwondo during competition. There was a significant difference (*p* < 0.001) between studies that reported sample size (IR = 2.51) and those that did not (IR = 0.91).

**Table 3:** Moderator analyses for subgroup values by athlete exposures

Moderator	Studies	Outcomes	IR	95% CI	*I*^2^	*p*
Sex	47	336	1.17	0.77–1.58	78.3%	0.157
Female	29	142	0.91	0.51–1.64	79.5%	
Male	36	194	1.46	0.91–2.36	77.3%	
Country	61	416	1.41	1.02–1.24	78.9%	< 0.001
Australia	2	7	2.10	0.25–17.78	44.4%	
Canada	3	33	5.52	1.26–24.16	61.4%	
Greece	1	4	3.63	1.15–11.47	68%	
South Korea	1	5	47.12	37.50–59.20	66.9%	
United States	54	367	1.12	0.85–1.48	10.1%	
Exposure	60	412	1.38	0.99–1.91	79%	0.207
Estimated	28	165	1.07	0.63–1.81	46.3%	
Exact	32	247	1.63	1.07–2.48	83.5%	
Setting	61	416	1.41	1.02–1.94	78.9%	< 0.001
Competition	47	170	2.39	1.72–3.30	86.9%	
Practice	37	138	0.51	0.37–0.68	0%	
Practice and competition	14	108	0.47	0.23–0.94	26.9%	
Level of contact	61	416	1.41	1.02–1.94	78.9%	< 0.001
Collision	46	188	1.72	1.27–2.32	47.1%	
Combat	11	26	1.01	0.28–3.63	88.3%	
Contact	17	92	0.89	0.47–1.69	18.2%	
Non-Contact	13	110	0.41	0.22–0.77	0%	
Reported sample size	61	416	1.41	1.02–1.94	78.9%	< 0.001
No	37	273	0.91	0.66–1.24	0%	
Yes	24	143	2.51	1.49–4.24	86.9%	

*p*-value < .05 indicates that there was a significant difference between subgroups

#### Incidence of Concussion by Player Hours

**Fig. 5:**
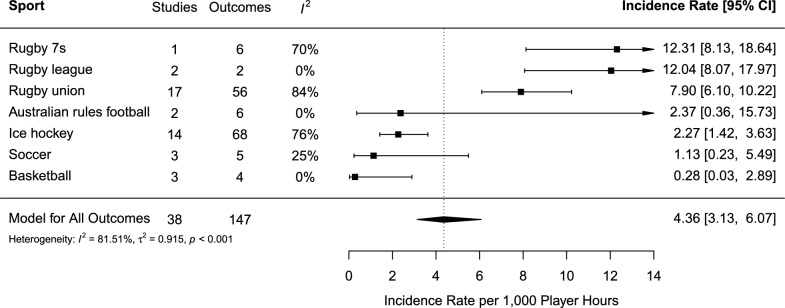
Forest plot of the sport-specific concussion incidence rates per 1000 player hours. *Note*: Three variables are truncated and indicated with arrowheads to the right of the plot. The arrowheads denote that confidence intervals extend beyond the plotted range

The pooled incidence of SRC per 1,000 PH across seven sports from 38 studies was 4.36 (95% CI 3.13–6.07) (Fig. 5). There was high (*I*^2^ = 82%) and significant (*p* < 0.001) heterogeneity in the pooled between-sport estimate. The incidence of SRC ranged from 0.28 (95% CI 0.03–2.89) for basketball to 12.31 (95% CI 8.13–18.64) for rugby 7s. Rugby league (IR = 12.04, 95% CI 8.07–17.97) and rugby union (IR = 7.90, 95% CI 6.10–10.22) also had very high incidence of SRC.

**Fig. 6:**
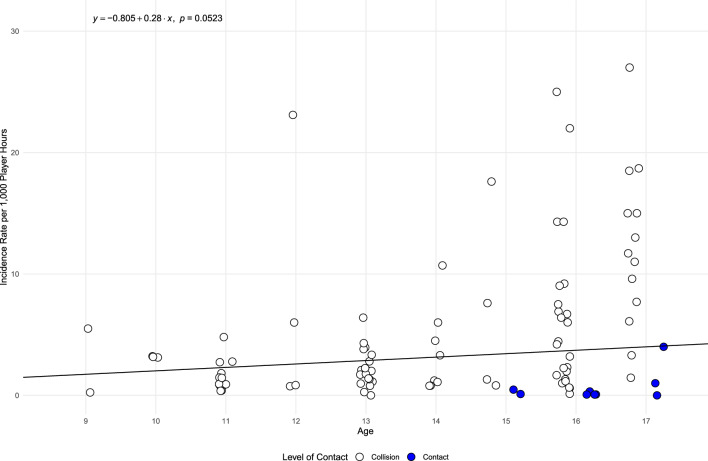
Bubble plot exploring the relationship between age and concussion incidence per 1000 player hours*. Note*: Points have a jitter along the x-axis to improve readability

**Fig. 7:**
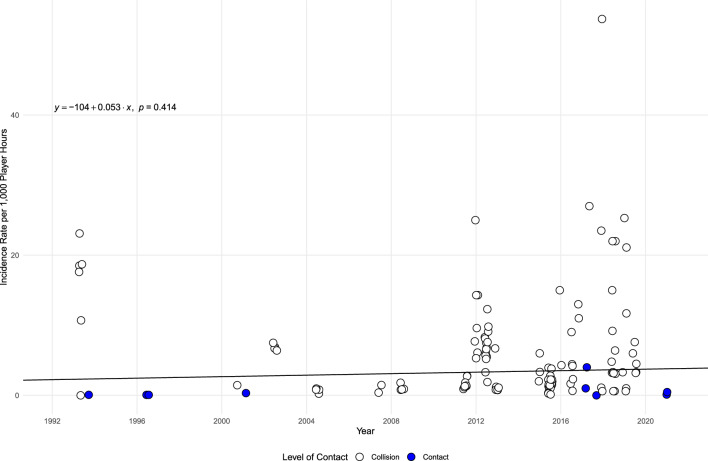
Bubble plot exploring the relationship between year and concussion incidence per 1000 player hours. *Note*: Points have a jitter along the x-axis to improve readability

The meta-regression analysis, as displayed in Fig. 6, shows a non-significant relationship between participant age and the incidence of SRC by PH (*p* = 0.052). The meta-regression analysis examining the relationship between the year and incidence of SRC by PH was not significant (*p* = 0.414), suggesting year did not moderate SRC incidence. However, as observed in Fig. 7, these results may have been skewed by a single study investigating ice hockey from the 1990s [162].

As presented in Table 4, moderator analyses found that within studies that reported concussion incidence by PH, there was no significant effect for sex (*p* = 0.533), exposure measurement (*p* = 0.886), country (*p* = 0.303), permitted or prohibited bodychecking in ice hockey (*p* = 0.407), or whether sample size was reported (*p* = 0.476). The results revealed that there was a significant difference in incidence according to the setting (*p* < 0.001), as competition was found to have the highest incidence (IR = 6.94). Level of contact was found to be a significant moderator of SRC incidence (*p* < 0.001), with collision sports having the highest incidence (IR = 4.77).

**Table 4:** Moderator analyses for subgroup values by player hours

Moderator	Studies	Outcomes	IR	95% CI	*I*^2^	*p*
Sex	23	70	5.88	4.04–8.55	84.0%	0.533
Female	8	15	5.74	2.33–14.16	88.7%	
Male	21	55	6.30	4.30–9.24	77.4%	
Country	36	146	4.21	2.98–5.96	81.9%	0.190
Australia	7	19	4.79	3.46–6.63	0%	
Canada	15	73	2.64	1.55–4.50	87.1%	
England	4	12	8.24	4.91–13.82	80.2%	
Ireland	3	5	6.73	3.91–11.57	55.4%	
New Zealand	1	2	1.10	0.25–4.81	0%	
South Africa	2	20	7.24	6.13–8.56	0%	
United States	4	15	14.19	10.75–18.72	60.7%	
Exposure	38	148	4.38	3.14–6.12	81.7%	0.886
Estimated	23	75	4.58	3.10–6.77	78.9%	
Exact	15	73	3.88	2.06–7.31	84.1%	
Setting	38	148	4.38	3.14–6.12	81.7%	< 0.001
Competition	23	74	6.94	4.82–10.00	82.6%	
Practice	5	10	0.67	0.30–1.49	0%	
Practice and Competition	15	64	2.18	1.22–3.90	76.9%	
Level of contact	38	148	4.38	3.14–6.12	81.7%	< 0.001
Collision	35	139	4.77	3.40–6.68	82.2%	
Contact	5	9	0.75	0.18–3.17	6.6%	
Body checking	5	12	2.37	1.61–3.51	0%	0.407
Permitted	5	6	2.73	1.64–4.55	0%	
Prohibited	5	6	1.95	1.06–3.58	0%	
Reported sample size	38	148	4.38	3.14–6.12	81.7%	0.476
No	4	7	6.75	4.78–9.54	21.4%	
Yes	34	141	4.14	2.84–6.03	82.3%	

*p-*value < 0.05 indicates that there was a significant difference between subgroups

#### Incidence of Concussion by Sex According to Sport

Moderator analyses comparing concussion incidence between males and females within sports are presented in Table 5. Sex was a significant moderator in soccer and rugby union, but it was not a significant moderator in basketball, softball/baseball, lacrosse, or ice hockey. In both soccer and rugby union, females had a higher concussion IR than males.

**Table 5:** Moderator analyses for sex according to sport

Moderator (incidence measurement)	Studies	Outcomes	IR	95% CI	*I*^2^	*p*
Soccer (AE)	13	41	0.93	0.52–1.68	35.7%	0.044
Female	12	23	1.23	0.63–2.43	47.1%	
Male	10	18	0.53	0.25–1.13	0%	
Basketball (AE)	10	34	0.54	0.29–1.02	0%	0.060
Female	9	17	0.80	0.39–1.66	0%	
Male	9	17	0.26	0.09–0.76	0%	
Softball/Baseball (AE)	9	28	0.37	0.16–0.83	0%	0.920
Female	8	15	0.38	0.14–1.03	0%	
Male	7	13	0.32	0.08–1.37	0%	
Lacrosse (AE)	16	61	0.76	0.52–1.10	0%	0.298
Female	11	23	0.58	0.31–1.09	0%	
Male	13	38	0.87	0.55–1.38	0%	
Rugby Union (PH)	15	36	7.24	5.19–10.09	86.8%	0.050
Female	2	5	10.41	2.16–50.19	90.0%	
Male	14	31	6.99	5.23–9.33	76.1%	
Ice Hockey (PH)	4	18	3.25	0.85–12.43	82.6%	0.130
Female	2	3	1.10	0.36–3.37	0%	
Male	4	15	3.34	0.86–13.03	82.3%	

AE, athlete exposure; PH, player hours. *p-*value < 0.05 indicates that there was a significant difference between subgroups

## Discussion

The primary aim of this systematic review and meta-analysis was to investigate the incidence of SRC in children and adolescents, and to assess how this differs across different sport types, age, and year of data collection. A secondary aim was to consider the impact of other potential moderating factors on SRC incidence rates, such as sex, country, setting, and level of contact. Overall, there were 116 studies included in the systematic review and 99 in the meta-analysis, with 65% of studies based in the USA. The pooled incidence of SRC per 1000 AE was found to be 1.41 across 21 sports, and 4.36 per 1000 PH across seven sports. The previous review included 23 studies, 13 of which were included in their meta-analysis [30]. One study from the previous review was not included in the current review as it could not be found, resulting in this review including 94 studies that were not included in the previous review. There were 10 studies, published prior to the September 2014 search date of the previous review, that were included in our review. All 13 studies from the previous meta-analysis were included, resulting in an additional 86 studies in our meta-analysis. This includes seven studies that were included in the previous review but were not included in their meta-analysis. Finally, our review includes an analysis of player hours in addition to athlete exposures. Consequently, this study offers a comprehensive and up-to-date review of the concussion risks associated with adolescent sports participation, which has important implications for youth athletes, parents, coaches, organisations, and policy makers.

For incidence measured by 1000 AE, the highest concussion incidence was found in taekwondo followed by rugby union, ice hockey, and American Football. For incidence measured by 1000 PH, the highest concussion incidence was found in rugby 7s, rugby league, and rugby union. Additionally, moderator analyses revealed that collision sports had higher SRC incidence compared to sports with lower-level contact. Therefore, it is clear that collision sports need to be the focus for interventions to reduce concussion risk [163] and consideration of equipment and rules changes to protect youth athletes [164, 165].

Prior research has suggested that females have a higher incidence of SRC across all ages, particularly in sports such as basketball and soccer [31]. We failed to find a significant difference in IR between males and females when considering all sports together; however, we did find some differences between sexes when considering individual sports. Given that sex-based differences have been found in our current meta-analysis and other studies not included in our meta-analysis [156, 160, 161], it is important to consider what may account for these differences and what could be done to modify concussion incidence. For example, contact rule differences between men and women’s high-school and college lacrosse result in females suffering fewer concussions than males [166, 167]. With the increase in female participation in collision sports such as Australian Rules football [168] and the rugby codes [169], it is important for policy makers to closely monitor SRC incidence, and to investigate factors that influence any sex-based differences in concussion incidence.

Moderator analyses revealed that there was no significant difference in incidence between estimated and exact exposure measurement techniques. This result highlights the value of cost-effective methods of exposure estimation for youth community teams and schools with limited resources. That is, an estimation of athlete exposure appears sufficient for athlete monitoring and reporting of SRC incidence. Additionally, sporting bodies and organisations may find this information useful as estimating exposure simplifies data collection processes, which can assist injury surveillance in becoming more efficient and accessible. While estimated exposure methods can be used to provide a simplified estimate of concussion risk, other approximation methods (e.g., Athletes-At-Risk method) may provide a more accurate and granular understanding that more closely matches individual exposure [35, 170]. It is recommended that the most exact measurement that resources allow be utilized, with a recognition that some measurement (even an estimation) will be more beneficial than no measurement.

Although the analysis was non-significant, it appears there may be a positive relationship between incidence (measured by PH) and age, indicating that as athletes mature, incidence of SRC may increase. Various sports that have a comparatively high risk of concussion (such as the rugby codes) may be influenced by the varying height and weight of youth athletes that arise from differences in child maturation and growth [171]. Additionally, rules in some sports change based on player age (e.g., bodychecking in youth ice hockey) which may also impact concussion risk as players who mature earlier may have advantages in size, strength, and speed [172–174]. Biological banding is a method that addresses the imbalance in biological maturation by grouping athletes based on growth and maturation attributes instead of age [175]. This approach has potential benefits, such as reducing the risk of injury, as indicated in previous research on youth soccer players in the UK [176]. Given these findings, the further promotion of the use of biological banding within these types of high contact sports may be worth considering, particularly in relation to addressing elevated SRC injury risks.

The overall incidence per 1000 AE was found to be larger than was observed in the previous review [30]. It is unclear exactly why this difference was found, although the current review includes a broad range of sports and substantially more papers than the previous review, which may account for this difference. However, the current study found that SRC incidence among youth did not change over time, which may be attributed to several factors. First, with the intention of reducing the risk of concussion, there has been an increase in the implementation of safety interventions and rules changes within various sports which have been shown to decrease incidence rates [166]. Second, there has been a growing awareness of SRC and the use of education programs and surveillance systems designeds to address the underreporting of concussion among youth, which have been shown to increase reporting rates [177]. It is possible that real concussion incidence has decreased while reporting rates have increased, resulting in a reported incidence rate that has not changed over time. However, it is important to consider that the current analysis used the median year of data collection for studies when they did not report incidence by year. This approach may have resulted in pooling incidence rates estimates across multiple years, contributing to an inaccurate estimate of SRC incidence over time. Therefore, future incidence studies should make data available for each collection year to allow accurate estimation of incidence over time.

Whilst the rugby codes, ice hockey, and American Football continued to have a high incidence of SRC [30], it is important to highlight the large incidence found in taekwondo (IR = 11.29). This may be attributed to all three taekwondo studies quantifying incidence during competition. As demonstrated in this review and previous research on adult athletes [178], athletes experience higher rates of SRC during competition in comparison to practice. Importantly, rule changes that award competitors more points for successful kicks to the head have led to an increase in the incidence and severity of head injuries [179, 180]. These rule changes took place after the data collection period of studies included in this review, and therefore it is important to consider the impact these changes have on future concussion incidence in taekwondo for youth athletes.

Our analyses revealed significant heterogeneity in the overall SRC estimate across sports. Although this heterogeneity was anticipated due to varying levels of contact and competition, the substantial heterogeneity limits the value of the overall IR and emphasises the importance of considering concussion incidence by specific sport. However, it is evident from our current analyses that despite the large number of studies included in the review, the number of outcomes for some sports is relatively few, and therefore these outcomes should be interpreted with caution. This review did not assess for publication bias across studies; however, this decision resulted from there being no established procedures for assessing publication bias within multi-level analyses of IR [181], and not assessing publication bias is common for this type of analysis [182]. Additionally, concussion incidence changes over time and according to athlete age within each sport were not explored, and therefore we are unable to comment on potential changes according to these factors within sports. Finally, given that the quality assessment tool used does not provide an overall rating of study quality, moderation analyses according to study quality could not be completed.

Several studies included in this review did not provide a clear concussion definition. Therefore, this lack of definition and the subtlety of several concussive symptoms may have led to a potential underestimation of the number of concussions. Although the included studies contributed a substantial total sample size, 40.5% of studies did not report sample size. Moderation analysis revealed larger IR for AE studies that reported sample sizes compared to studies that did not report sample size, but this finding was not reflected in PH studies. Regardless, not reporting sample size is a concerning trend that all future researchers should strive to correct. Additionally, numerous studies did not specify the participants' sex (e.g., studies exploring American Football and rugby union) or age information, and therefore, despite the existence of useful IR data, these data could not be included as part of the sex or age analyses.

Future research must continue to examine the effectiveness of injury prevention strategies intended to reduce SRC incidence, particularly in relation to competition, whereby incidence is higher than in practice. Future intervention and education efforts should consider awareness of concussion symptoms and strategies to improve disclosure of suspected concussion among young athletes, as underreporting is a common issue [183]. Further, research should explore the efficacy of these education programs on youth mental health outcomes and consider longitudinal success (i.e., knowledge retention) in addition to immediate concussion knowledge and incidence outcomes following intervention [184].

It is anticipated that this study can serve as a valuable resource for youth athletes, families, and coaches in understanding the potential concussion risks associated with participation in sport. For example, by having a clear understanding of the difference in concussion risk between collision and (non-)contact sports, participants can make a more informed decision about their own safety while participating in sport. Further, our findings may inform specific sporting bodies (e.g., those that are classified as collision sports) of the risk of participation for youth athletes, and consequently the importance of reducing SRC incidence. Finally, our findings may provide value to public health officials when considering targeted funding towards youth SRC risk reduction.

## Conclusions

It is crucial that athletes, parents, coaches, and healthcare providers understand the degree of concussion risk associated with participating in specific sports. This systematic review and meta-analysis provides updated insight into the incidence of concussion for youth athletes across 28 sports. Collision sports such as the rugby codes, American Football, and ice hockey put youth athletes at a much higher risk of concussion than sports with lower levels of contact, and concussion incidence is markedly higher during competition than in practice. Sport organizations and governing bodies may find an estimated exposure measurement to be a cost-effective and time efficient method of understanding concussion incidence in their specific setting, but should be aware of the potential limitations of such measures.

## Supplementary Information

Supplementary file 1

## Data Availability

The datasets used and/or analysed during the current study are available from the corresponding author on reasonable request.

## Abbreviations

AE: Athlete exposure
CTE: Chronic traumatic encephalopathy
IR: Incidence rate
PERSiST: PRISMA in exercise, rehabilitation, sport medicine and sports science
PH: Player hours
PRISMA: Preferred reporting items for systematic reviews and meta-analyses
SRC: Sport-related concussions
