# Supplementary material for: The Incidence of Sports-Related Concussion in Children and Adolescents: A Systematic Review and Meta-Analysis
Source: Sports Med Open. 2025 Apr 11;11:36. doi: 10.1186/s40798-025-00834-9 (PMC11992322; doi:10.1186/s40798-025-00834-9)
Supplement: Supplementary file 1 [file 40798_2025_834_MOESM1_ESM.docx]

**Supplementary Material**

**The Incidence of Sports-Related Concussion in Children and Adolescents: A Systematic Review and Meta-Analysis**

**Sports Medicine - Open**

Veronica Ingram^1, 2^, Megan Fielding^1, 2^, Laura AM Dunne^1, 3^, Stefan Piantella^1, 2^, Jonathon Weakley^1, 3, 4^, Rich D Johnston^1, 3, 4^, Thomas B McGuckian*^1, 2^

^1^ School of Behavioural and Health Sciences, Australian Catholic University, Australia

^2^ Healthy Brain and Mind Research Centre (HBMRC), Australian Catholic University, Australia

^3^ Sports Performance, Recovery, Injury and New Technologies (SPRINT) Research Centre, Australian Catholic University, Australia

^4^ Carnegie Applied Rugby Research (CARR) Centre, Carnegie School of Sport, Leeds Beckett University, United Kingdom

*Corresponding author:

Thomas B McGuckian

Australian Catholic University

115 Victoria Parade, Fitzroy, Melbourne, VIC 3065, Australia

Email: Thomas.mcguckian@acu.edu.au

Tel: +61 3 9230 8323

**Supplementary Material A**

**Database Search Strategies Across Databases**

**Database Search Strategy: Medline**

S1. TI (concussion* OR “brain injur*” OR “head injur*” OR TBI OR mTBI OR “head impact*”) OR AB (concussion* OR “brain injur*” OR “head injur*” OR TBI OR mTBI OR “head impact*”)

S2. TI (sport OR sports OR athlete* OR football OR rugby OR hockey OR lacrosse OR soccer OR basketball OR baseball OR softball OR wrestling OR track OR taekwondo OR volleyball OR cheerleading OR netball OR cricket OR cycling OR gymnast* OR motorcross OR sailing OR snowboarding OR skiing OR skateboarding OR skating OR luge OR skeleton OR bobsleigh) OR AB ( sport OR sports OR athlete* OR football OR rugby OR hockey OR lacrosse OR soccer OR basketball OR baseball OR softball OR wrestling OR track OR taekwondo OR volleyball OR cheerleading OR netball OR cricket OR cycling OR gymnast* OR motorcross OR sailing OR snowboarding OR skiing OR skateboarding OR skating OR luge OR skeleton OR bobsleigh)

S3. TI (adolescent* OR youth OR child* OR school OR student* OR paediatric* OR pediatric* OR minor* OR teen*) OR AB (adolescent* OR youth OR child* OR school OR student* OR paediatric* OR pediatric* OR minor* OR teen*)

S4. TI (prevalence OR “cohort stud*” OR “cross sectional stu*” OR “follow-up stud*” OR incidence) OR AB (prevalence OR “cohort stud*” OR “cross sectional stu*” OR “follow-up stud*” OR incidence)

S5. (MH "Brain Concussion")

S6. (MH "Brain Injuries")

S7. (MH "Craniocerebral Trauma")

S8. S5 OR S6 OR S7

S9. (MH "Sports")

S10. (MH "Athletes")

S11. S9 OR S10

S12. (MH "Adolescent")

S13. (MH "Child")

S14. (MH "Schools")

S15. (MH "Students")

S16. (MH "Pediatrics")

S17. (MH "Minors")

S18. S12 OR S13 OR S14 OR S15 OR S16 OR S17

S19. (MH "Prevalence")

S20. (MH "Cohort Studies")

S21. (MH "Cross-Sectional Studies")

S22. (MH "Follow-Up Studies")

S23. (MH "Incidence")

S24. S19 OR S20 OR S21 OR S22 OR S23

S25. S1 OR S8

S26. S2 OR S11

S27. S3 OR S18

S28. S4 OR S24

S29. S25 AND S26 AND S27 AND S28

**Database Search Strategy: PsycInfo**

S1. TI ( concussion* OR “brain injur*” OR “head injur*” OR TBI OR mTBI OR “head impact*” ) OR AB ( concussion* OR “brain injur*” OR “head injur*” OR TBI OR mTBI OR “head impact*” )

S2. TI ( sport OR sports OR athlete* OR football OR rugby OR hockey OR lacrosse OR soccer OR basketball OR baseball OR softball OR wrestling OR track OR taekwondo OR volleyball OR cheerleading OR netball OR cricket OR cycling OR gymnast* OR motorcross OR sailing OR snowboarding OR skiing OR skateboarding OR skating OR luge OR skeleton OR bobsleigh ) OR AB ( sport OR sports OR athlete* OR football OR rugby OR hockey OR lacrosse OR soccer OR basketball OR baseball OR softball OR wrestling OR track OR taekwondo OR volleyball OR cheerleading OR netball OR cricket OR cycling OR gymnast* OR motorcross OR sailing OR snowboarding OR skiing OR skateboarding OR skating OR luge OR skeleton OR bobsleigh )

S3. TI ( adolescent* OR youth OR child* OR school OR student* OR paediatric* OR pediatric* OR minor* OR teen* ) OR AB ( adolescent* OR youth OR child* OR school OR student* OR paediatric* OR pediatric* OR minor* OR teen* )

S4. TI ( prevalence OR “cohort stud*” OR “cross sectional stu*” OR “follow-up stud*” OR incidence ) OR AB ( prevalence OR “cohort stud*” OR “cross sectional stu*” OR “follow-up stud*” OR incidence )

S5. ((DE "Brain Concussion") OR (DE "Brain Injuries")) OR (DE "Head Injuries")

S6. (DE "Sports") OR (DE "Athletes")

S7. ((((DE "Child Health") OR (DE "Adolescent Health")) OR (DE "Students")) OR (DE "Schools")) OR (DE "Pediatrics")

S8. DE "Followup Studies"

S9. S1 OR S5

S10. S2 OR S6

S11. S3 OR S7

S12. S4 OR S8

S13. S9 AND S10 AND S11 AND S12

**Database Search Strategy: SportsDiscus**

**S1.** TI ( concussion* OR “brain injur*” OR “head injur*” OR TBI OR mTBI OR “head impact*” ) OR AB ( concussion* OR “brain injur*” OR “head injur*” OR TBI OR mTBI OR “head impact*” )

**S2.** ((DE "BRAIN concussion") OR (DE "BRAIN injuries")) OR (DE "HEAD injuries")

**S3.** TI ( Sport OR Sports OR Athlete* OR football OR rugby OR hockey OR lacrosse OR soccer OR basketball OR baseball OR softball OR wrestling OR track OR taekwondo OR volleyball OR cheerleading OR netball OR cricket OR cycling OR gymnast*or motorcross or sailing or snowboarding or skiing or skateboarding or skating ) OR AB ( Sport OR Sports OR Athlete* OR football OR rugby OR hockey OR lacrosse OR soccer OR basketball OR baseball OR softball OR wrestling OR track OR taekwondo OR volleyball OR cheerleading OR netball OR cricket OR cycling OR gymnast* OR motorcross OR sailing OR snowboarding OR skiing OR skateboarding OR skating OR luge OR skeleton OR bobsleigh)

**S4.** (DE "SPORTS") OR (DE "ATHLETES")

**S5.** TI ( Adolescent* OR youth OR Child* OR School OR Student* OR Paediatric* OR Pediatric* OR minor* OR teen* ) OR AB ( Adolescent* OR youth OR Child* OR School OR Student* OR Paediatric* OR Pediatric* OR minor* OR teen* )

**S6.** (((((((DE "ADOLESCENT health") OR (DE "YOUTH")) OR (DE "CHILDREN'S health")) OR (DE "CHILDREN")) OR (DE "SCHOOLS")) OR (DE "STUDENTS")) OR (DE "PEDIATRICS")) OR (DE "TEENAGERS")

**S7.** TI ( Prevalence OR “Cohort Stud*” OR “Cross Sectional Stu*” OR “Follow-up Stud*” OR Incidence ) OR AB ( Prevalence OR “Cohort Stud*” OR “Cross Sectional Stu*” OR “Follow-up Stud*” OR Incidence )

**S8.** S1 OR S2

**S9.** S3 OR S4

**S10.** S5 OR S6

**S11.** S7 AND S8 AND S9 AND S10

**Database Search Strategy: Web of Science**

**S1.** TI(concussion* OR “brain injur*” OR “head injur*” OR TBI OR mTBI OR “head impact*”) OR AB (concussion* OR “brain injur*” OR “head injur*” OR TBI OR mTBI OR “head impact*”))

**S2.** TI (sport OR sports OR athlete* OR football OR rugby OR hockey OR lacrosse OR soccer OR basketball OR baseball OR softball OR wrestling OR track OR taekwondo OR volleyball OR cheerleading OR netball OR cricket OR cycling OR gymnast* OR motorcross OR sailing OR snowboarding OR skiing OR skateboarding OR skating OR luge OR skeleton OR bobsleigh) OR AB ( sport OR sports OR athlete* OR football OR rugby OR hockey OR lacrosse OR soccer OR basketball OR baseball OR softball OR wrestling OR track OR taekwondo OR volleyball OR cheerleading OR netball OR cricket OR cycling OR gymnast* OR motorcross OR sailing OR snowboarding OR skiing OR skateboarding OR skating OR luge OR skeleton OR bobsleigh)

**S3.** TI (adolescent* OR youth OR child* OR school OR student* OR paediatric* OR pediatric* OR minor* OR teen*) OR AB ( adolescent* OR youth OR child* OR school OR student* OR paediatric* OR pediatric* OR minor* OR teen* )

**S4.** TI ( prevalence OR “cohort stud*” OR “cross sectional stu*” OR “follow-up stud*” OR incidence ) OR AB ( prevalence OR “cohort stud*” OR “cross sectional stu*” OR “follow-up stud*” OR incidence )

**S5.** S1 AND S2 AND S3 AND S4

**Database Search Strategy: Embase**

**S1.** (concussion* OR “brain injur*” OR “head injur*” OR TBI OR mTBI OR “head impact*”). ti OR (concussion* OR “brain injur*” OR “head injur*” OR TBI OR mTBI OR “head impact*”).ab

**S2.** concussion/

**S3.** head injury/

**S4.** brain injury/

**S5.** S2 OR S3 OR S4

**S6.** S1 OR S5

**S7.** (sport OR sports OR athlete* OR football OR rugby OR hockey OR lacrosse OR soccer OR basketball OR baseball OR softball OR wrestling OR track OR taekwondo OR volleyball OR cheerleading OR netball OR cricket OR cycling OR gymnast* OR motorcross OR sailing OR snowboarding OR skiing OR skateboarding OR skating OR luge OR skeleton OR bobsleigh).ti. OR (sport OR sports OR athlete* OR football OR rugby OR hockey OR lacrosse OR soccer OR basketball OR baseball OR softball OR wrestling OR track OR taekwondo OR volleyball OR cheerleading OR netball OR cricket OR cycling OR gymnast* OR motorcross OR sailing OR snowboarding OR skiing OR skateboarding OR skating OR luge OR skeleton OR bobsleigh).ab.

**S8.** sport/

**S9.** athlete/

**S10.** S8 OR S9

**S11.** S7 OR S10

**S12.** (adolescent* OR youth OR child* OR school OR student* OR paediatric* OR pediatric* OR minor* OR teen*).ti. OR (adolescent* OR youth OR child* OR school OR student* OR paediatric* OR pediatric* OR minor* OR teen*).ab.

**S13.** adolescent/

**S14.** child/

**S15**. school/

**S16.** student/

**S17**. pediatrics/

**S18.** S13 OR S14 O4 S15 OR S16 OR S17

**S19.** S12 OR S18

**S20.** (prevalence OR “cohort stud*” OR “cross sectional stu*” OR “follow-up stud*” OR incidence).ti. OR (prevalence OR “cohort stud*” OR “cross sectional stu*” OR “follow-up stud*” OR incidence).ab.

**S21.** prevalence/

**S22.** cross-sectional study/

**S23.** follow up/

**S24.** incidence/

**S25.** S21 OR S22 OR S23 OR S24

**S26.** S20 OR S25

**S27.** S6 AND S11 AND S19 AND S26

**Supplementary Material B**

**Study Quality Assessment**

| Cohort Details | | Cohort Selection and Measurement | | Analysis | Outcome Measurement | | | | | |
| --- | --- | --- | --- | --- | --- | --- | --- | --- | --- | --- |
| Study | Sport | Representativeness of Exposed Cohort | Exposure Measurement | Study Results Stratified by Important Factors | Outcome Ascertainment | Concussion Defined | Mechanism of Injury | Study Follow-Up | Previous History Reported | Practice and Competition |
| Ali (2023) | American Football, Soccer, Basketball, Lacrosse, Gymnastics, Cheerleading, Wrestling, Boxing, Martial Arts, Water Polo, Diving, Baseball, Softball, Volleyball, and Track and Field. | Yes | Patient years | No | Physician or trainer | Yes | No | 10 years | Yes | Yes |
| Ali (2021a) | American Football, Soccer, Basketball, Volleyball, Lacrosse, Baseball, Softball, Cheerleading and Wrestling. | Yes | Patient years | No | Physician or trainer | Yes | No | 10 years | Yes | Yes |
| Ali (2021b) | American Football, Lacrosse, Wrestling, Ice Hockey, Soccer, Basketball, Volleyball, Baseball, Softball, and Cheerleading. | Yes | Patient years | No | Physician or trainer | Yes | No | 10 years | Yes | Yes |
| Archbold (2017) | Rugby Union | Yes | PH | No | Physiotherapist, emergency medical practitioner, physician, nurse, coach, parent, or self-diagnosed | No | Yes | 1 season | Yes | Yes |
| Barden (2021) | American Football, Basketball, Soccer, Rugby Union, and Rugby League | Yes | PH | Yes | Medical staff | No | Yes | 5 years | No | Yes |
| Baron (2020) | Lacrosse | Yes | AE | No | Trainer | No | No | 2 seasons | No | Yes |
| Beis (2007) | Taekwondo | Yes | AE | Yes | Physician | No | Yes | NR | No | No |
| Black (2016) | Ice Hockey | Yes | PH | No | Physiotherapist or athletic therapist | Yes | Yes | 1 season | Yes | Yes |
| Blake (2018) | Ice Hockey | Yes | PH | Yes | Physician | Yes | No | 3 seasons | Yes | Yes |
| Bretzin (2018) | Basketball, Baseball, American Football, Ice Hockey, Lacrosse, Soccer, Swimming and Diving, Wrestling, Cheerleading, Softball and Volleyball | Yes | Player seasons | Yes | Trainer, administrator, or coach | Yes | Yes | 1 year | Yes | Yes |
| Bretzin (2021) | Soccer | Yes | Athlete seasons | Yes | Trainer, coach, or school official | Yes | Yes | 2 years | Yes | Yes |
| Bretzin (2024) | American Football, Basketball, Soccer, Wrestling, Volleyball, Cheerleading, Lacrosse, Softball, Ice Hockey, Baseball, Swimming and Diving, Track and Field, Gymnastics, Tennis, Cross-country, Golf, Water Polo, Field Hockey, Bowling | Yes | Player seasons | Yes | Medical staff | Yes | Yes | 8 years | No | Yes |
| Clifton (2018a) | Basketball | Yes | AE | No | Trainer | No | Yes | 9 years | No | Yes |
| Clifton (2018b) | Basketball | Yes | AE | No | Trainer | No | Yes | 9 years | No | Yes |
| Collins (2016) | American Football | Yes | AE | No | Trainer or physician | No | No | 5 seasons | No | Yes |
| Cosgrave (2023) | Rugby Union | Yes | PH | Yes | Team physiotherapist or physician | No | No | 1 year | Yes | Yes |
| Covassin (2018) | American Football, Ice Hockey, Soccer, Basketball, Wrestling, Cheerleading, Lacrosse, Volleyball, Softball, and Baseball. | Yes | Per 100 athletes | Yes | Trainer, coach or school official | Yes | No | 1 year | No | Yes |
| DeLee (1992) | American Football | Yes | Players | No | Trainer | No | No | 1 season | No | Yes |
| DiStefano (2018) | Soccer | Yes | AE | No | Trainer or physician | No | Yes | 10 years | No | Yes |
| Dompier (2015) | American Football | Yes | AE | Yes | Trainer | Yes | No | 2 seasons | No | Yes |
| Dugan (2014) | Basketball, Cheerleading, American Football, Ice Hockey, Soccer, Volleyball, Gymnastics, Lacrosse, Baseball, Dance, Cross Country and Wrestling. | Yes | Per 100 athletes | Yes | Trainer | No | No | 1 year | No | Unspecified |
| Echlin (2010) | Ice Hockey | Yes | AE | No | Physician and non-physician observer | Yes | No | 1 season | Yes | No |
| Eliason (2022a) | Ice Hockey | Yes | PH | No | Athletic therapist or physiotherapist | Yes | No | 2 seasons | Yes | No |
| Eliason (2022b) | Ice Hockey | Yes | PH | No | Physician or athletic therapist | Yes | No | 3 seasons | Yes | No |
| Eliason (2023) | Ice Hockey | Yes | PH | Yes | Athletic therapist or physiotherapist | Yes | No | 5 years | Yes | Yes |
| Emery (2022) | Ice Hockey | Yes | PH | No | Safety coach, manager, and athletic therapist | Yes | Yes | 3 seasons | Yes | No |
| Emery (2010) | Ice Hockey | Yes | PH | No | Athletic therapist | Yes | Yes | 1 season | Yes | Yes |
| Emery (2006) | Ice Hockey | Yes | PH | Yes | Athletic therapist | No | Yes | 1 season | Yes | Yes |
| Emery (2011) | Ice Hockey | Yes | PH | Yes | Athletic therapist or physiotherapist | Yes | Yes | 1 season | Yes | Yes |
| Emery (2020) | Ice Hockey | Yes | PH | No | Manager or athletic therapist | Yes | Yes | 2 seasons | Yes | No |
| Fremont (2022) | Canadian Football | Yes | AE | Yes | Physiotherapist or physician | Yes | No | 4 seasons | No | Yes |
| Gessel (2007) | American Football, Basketball, Volleyball, Soccer/Football, Wrestling, Baseball, and Softball | Yes | AE | Yes | Trainer | Yes | Yes | 1 season | No | Yes |
| Gomez (1996) | Basketball | Yes | PH | No | Trainer | No | No | 1 season | No | Yes |
| Guillaume (2021) | Lacrosse | Yes | AE | No | Trainer | No | Yes | 9 seasons | No | Yes |
| Guskiewicz (2000) | American Football | Yes | AE | No | Trainer or physician | Yes | Yes | 3 seasons | Yes | Yes |
| Hancock (2024) | Rugby Union | Yes | PH | Yes | Coach or therapist | Yes | Yes | 3 seasons | No | No |
| Hannah (2021) | American Football and other sports | Yes | Patient years | Yes | Qualified member of athletic staff | Yes | No | 10 years | Yes | Unspecified |
| Haseler (2010) | Rugby Union | Yes | PH | Yes | Coach or first aider | Yes | No | 1 season | No | No |
| Hecimovich (2017) | Australian Rules Football | Yes | AE | Yes | Designated representative and physician | N | Yes | 1 season | No | No |
| Herman (2022) | Lacrosse | Yes | AE | No | Trainer, physician or allied health | No | Yes | 3 seasons | No | Yes |
| Hinton (2005) | Lacrosse | Yes | AE | Yes | Trainer | No | Yes | 3 seasons | No | Yes |
| Junge (2004) | Soccer and Rugby Union | Yes | PH | No | Physician | No | No | 1 season | No | Yes |
| Kawasaki (2023) | Rugby Union | Yes | PH | Yes | Physician | No | Yes | 1 season | No | No |
| Kerr (2016a) | Lacrosse | Yes | AE | Yes | Trainer | No | No | 1 season | No | Yes |
| Kerr (2019a) | American Football, Ice Hockey, Lacrosse, Wrestling, Soccer, Basketball, Baseball Swimming, Track and Field, Cross Country, Volleyball, Field Hockey, and Softball | Yes | AE | Yes | Trainer | Yes | Yes | 5 years | No | Yes |
| Kerr (2019b) | American Football | Yes | AE | No | Trainer | No | Yes | 3 years | No | Yes |
| Kerr (2017a) | Baseball, Basketball, American Football, Soccer, Track, Wrestling, Cheerleading, Softball, and Volleyball | Yes | AE | Yes | Trainer | Yes | No | 1 season | No | Yes |
| Kerr (2016b) | American Football | Yes | AE | No | Trainer or physician | No | Yes | 1 season | No | Yes |
| Kerr (2018a) | Volleyball | Yes | AE | No | Trainer | No | Yes | 9 years | No | Yes |
| Kerr (2015a) | American Football | Yes | AE | No | Trainer | Yes | No | 2 seasons | No | Yes |
| Kerr (2021) | Ice Hockey | Yes | AE | No | Trainer | No | Yes | 9 years | No | Yes |
| Kerr (2018b) | Soccer | Yes | AE | No | Trainer | No | Yes | 9 years | No | Yes |
| Kerr (2019c) | Lacrosse | Yes | AE | Yes | Trainer | No | Yes | 3 seasons | No | Yes |
| Kerr (2018c) | American Football | Yes | AE | No | Trainer | No | Yes | 9 years | No | Yes |
| Kerr (2015b) | American Football | Yes | AE | Yes | Trainer | No | No | 1 season | No | Yes |
| Kerr (2017b) | American Football | Yes | AE | Yes | Trainer | Yes | No | 1 season | No | No |
| Kerr (2016c) | American Football | Yes | AE | Yes | Trainer | Yes | No | 2 seasons | No | Yes |
| Koh (2004) | Taekwondo | Yes | AE | Yes | Trainer | Yes | No | 1 tournament | Yes | No |
| Kolstad (2023) | Ice Hockey | Yes | PH | Yes | Manager, coach, parent or athletic therapist | Yes | Yes | 5 seasons | Yes | Yes |
| Kontos (2013) | American Football | Yes | AE | Yes | Physician | Yes | Yes | 1 season | Yes | Yes |
| Kontos (2016) | Ice Hockey | YES | AE | Yes | Medical staff, athletic trainer or physician | Yes | Yes | 2 seasons | Yes | Yes |
| Kroshus (2018) | Wrestling | Yes | AE | No | Trainer | No | Yes | 9 years | No | Yes |
| Leahy (2023) | Rugby Union | Yes | PH | No | Teacher or coach | Yes | Yes | 2 seasons | No | Yes |
| Leung (2017a) | Rugby Union | Yes | PH | No | First aider or trainer | Yes | No | 1 season | No | No |
| Leung (2017b) | Rugby Union | Yes | PH | No | Trainer or physiotherapist | Yes | No | 1 season | No | No |
| Lincoln (2011) | American Football, Lacrosse, Soccer, Wrestling, Basketball, Baseball, Softball, Field Hockey and Cheerleading. | Yes | AE | Yes | Trainer | Yes | No | 11 seasons | No | Yes |
| Lincoln (2007) | Lacrosse | Yes | AE | Yes | Trainer | No | No | 4 seasons | No | Yes |
| Lincoln (2014) | Lacrosse | Yes | AE | Yes | Trainer | Yes | Yes | 1 season | No | No |
| Lopez (2020) | Rugby 7s | Yes | PH | Yes | Trainer or healthcare provider | No | No | 5 years | No | No |
| Lynall (2018b) | Ice Hockey | Yes | AE | No | Trainer | No | Yes | 6 years | No | Yes |
| Lynall (2018a) | Field Hockey | Yes | AE | No | Trainer | No | Yes | 6 years | No | Yes |
| Makovec Knight (2022) | Australian Rules Football | Yes | PH | Yes | Volunteer parent or trainer | Yes | No | 2 seasons | Yes | No |
| Marshall (2001) | Rugby Union | Yes | AE | No | Trainer | No | No | 3 seasons | No | No |
| Marshall (2015) | American Football, Soccer and Lacrosse | Yes | AE | Yes | Trainer | Yes | Yes | 3 years | Yes | Yes |
| McFie (2016) | Rugby Union | Yes | PH | Yes | Tournament doctor | Yes | Yes | 4 tournaments | No | No |
| McGinnis (2020) | Lacrosse | Yes | AE | No | Trainer | No | Yes | 1 season | No | No |
| McGuine (2014) | American Football | Yes | AE | No | Trainer | Yes | Yes | 2 seasons | Yes | Yes |
| McIntosh (2001) | Rugby Union | Yes | AE | No | Physician | No | Yes | 1 season | No | No |
| McIntosh (2009) | Rugby Union | Yes | AE | No | Recording officer or physician | Yes | No | 2 seasons | No | No |
| McMahon (1993) | Australian Rules Football | Yes | PH | Yes | Study coordinator | No | No | 1 season | No | Yes |
| Messina (1999) | Basketball | Yes | PH | Yes | Trainer | No | No | 1 season | No | Yes |
| Meyers (2019) | American Football | Yes | Team games | No | Trainer | Yes | Yes | 5 seasons | No | No |
| Meyers (2004) | American Football | Yes | Team games | No | Trainer or physician | No | Yes | 5 seasons | No | No |
| Morrissey (2022) | Ice Hockey | Yes | Per 10,000 athletes | Yes | Emergency department records | No | Yes | 18 years | No | No |
| Murray-Smith (2022) | Rugby Union | Yes | PH | Yes | Trainer | No | Yes | 3 seasons | No | Yes |
| O'Connor (2017) | American Football, Wrestling, Field Hockey, Gymnastics, Volleyball, Baseball, Softball, Basketball, Crew/Rowing, Cross-Country, Golf, Lacrosse, Soccer, Indoor Track and Field, Outdoor Track and Field, Swimming and Diving and Tennis | Yes | AE | Yes | Trainer or health care professional | Yes | Yes | 2 years | No | Yes |
| O'Kane (2014) | Soccer | Yes | AE | Yes | Parent report | Yes | Yes | 4 seasons | Yes | Yes |
| Peek (2023) | Soccer | Yes | PH | Yes | Physician | No | No | 1 season | No | No |
| Peterson (2017) | American Football | Yes | AE | No | Volunteer parent or trainer | Yes | No | 1 season | No | Yes |
| Pfaller (2019) | American Football | Yes | AE | No | Trainer | Yes | No | 3 seasons | Yes | Yes |
| Pierpoint (2019a) | Lacrosse | Yes | AE | Yes | Trainer, physician or health care professional | No | Yes | 6 years | No | Yes |
| Pierpoint (2019b) | Lacrosse | Yes | AE | Yes | Trainer, physician or health care professional | No | Yes | 6 years | No | Yes |
| Pieter (1999) | Taekwondo | Yes | AE | Yes | Physician | No | Yes | 3 tournaments | No | No |
| Powell (1999) | Baseball, Basketball, American Football, Soccer, Wrestling, Field Hockey, Softball and Volleyball | Yes | AE | Yes | Trainer | Yes | No | 3 seasons | No | Yes |
| Rivara (2014) | American Football, Soccer | Yes | AE | Yes | Self- or parent report | Yes | No | 1 season | Yes | Yes |
| Roberts (1999) | Ice Hockey | Yes | AE | Yes | Trainer | No | Yes | 4 tournaments | No | No |
| Schneider (2021) | Ice Hockey | Yes | PH | Yes | Physician | Yes | No | 1 season | Yes | Yes |
| Schulz (2004) | American Football, Soccer, Wrestling, Basketball, Softball, Baseball, Track and Cheerleading. | Yes | AE | Yes | Trainer | Yes | No | 1-8 seasons | Yes | Yes |
| Sewry (2018) | Rugby Union | Yes | PH | No | Doctor | No | Yes | 6 tournaments | No | No |
| Shill (2022) | Rugby Union | Yes | PH | Yes | Manager, coach, sports medicine class student, athletic therapist, physiotherapist or physician | No | Yes | 2 years | Yes | Yes |
| Shill (2024) | Rugby Union | Yes | PH | Yes | Physician | Yes | Yes | 2 years | Yes | Yes |
| Smith (2013) | American Football, Soccer, Basketball, Wrestling, Baseball, Volleyball, Softball, Ice Hockey, Lacrosse, Swimming and Diving, Cheerleading and Track and Field. | Yes | AE | No | Trainer | No | No | 7 seasons | No | Yes |
| Snyder Valier (2020) | Softball | Yes | AE | No | Trainer | No | No | 3 seasons | No | Yes |
| Spiera (2021) | American Football and other sports | Yes | Person years | Yes | Trainer, physician, coach or other faculty member | Yes | No | 11 years | Yes | Unspecified |
| Tee (2019) | Rugby League | Yes | AE | No | Physiotherapist | No | No | 1 season | No | No |
| Tisano (2022) | Gymnastics | Yes | Athlete days per year | Yes | Emergency department records | No | No | 7 years | No | Unspecified |
| Tuominen (2017) | Ice Hockey | Yes | PH | No | Physician or medical supervisor | No | Yes | 9 years | No | No |
| Vaandering (2022) | Volleyball | Yes | AE | Yes | Medical staff | Yes | Yes | 1 tournament | Yes | No |
| Warner (2018) | Lacrosse | Yes | AE | Yes | Trainer | No | No | 8 years | No | Yes |
| Wasserman (2019) | Softball | Yes | AE | No | Trainer | No | Yes | 9 years | No | Yes |
| West (2023) | Rugby Union | Yes | PH | No | Research assistant or study therapist | Yes | Yes | 2 seasons | Yes | Yes |
| Williams (2022) | Volleyball | Yes | AE | No | Trainer | No | Yes | 5 years | No | Yes |
| Yard (2009) | American Football, Soccer, Basketball, Wrestling, Baseball, Volleyball, Softball | Yes | AE | Yes | Trainer | Yes | No | 3 years | No | Yes |
| Zemper (2003) | American Football | Yes | AE | No | Trainer | Yes | No | 2 seasons | Yes | Yes |
| Zendler (2021) | Basketball, American Football and Soccer | Yes | Participant years | Yes | Emergency department records | No | Yes | 5 years | No | No |
| Zynda (2022) | Basketball | Yes | Participant years | Yes | Emergency department records | No | No | 7 years | No | No |

*Note.* Abbreviations: AE = Athlete Exposures; PH = Player Hours.
